# Stabilizing Genetically Unstable Simple Sequence Repeats in the Campylobacter jejuni Genome by Multiplex Genome Editing: a Reliable Approach for Delineating Multiple Phase-Variable Genes

**DOI:** 10.1128/mBio.01401-21

**Published:** 2021-08-24

**Authors:** Shouji Yamamoto, Sunao Iyoda, Makoto Ohnishi

**Affiliations:** a Department of Bacteriology I, National Institute of Infectious Diseases, Tokyo, Japan; Department of Veterinary Medicine

**Keywords:** *Campylobacter*, genome editing, natural transformation, phase variation, phenotypic engineering, simple sequence repeats

## Abstract

Hypermutable simple sequence repeats (SSRs) are major drivers of phase variation in Campylobacter jejuni. The presence of multiple SSR-mediated phase-variable genes encoding enzymes that modify surface structures, including capsular polysaccharide (CPS) and lipooligosaccharide (LOS), generates extreme cell surface diversity within bacterial populations, thereby promoting adaptation to selective pressures in host environments. Therefore, genetically controlling SSR-mediated phase variation can be important for achieving stable and reproducible research on C. jejuni. Here, we show that natural “cotransformation” is an effective method for C. jejuni genome editing. Cotransformation is a trait of naturally competent bacteria that causes uptake/integration of multiple different DNA molecules, which has been recently adapted to multiplex genome editing by natural transformation (MuGENT), a method for introducing multiple mutations into the genomes of these bacteria. We found that cotransformation efficiently occurred in C. jejuni. To examine the feasibility of MuGENT in C. jejuni, we “locked” different polyG SSR tracts in strain NCTC11168 (which are located in the biosynthetic CPS/LOS gene clusters) into either the ON or OFF configurations. This approach, termed “MuGENT-SSR,” enabled the generation of all eight edits within 2 weeks and the identification of a phase-locked strain with a highly stable type of Penner serotyping, a CPS-based serotyping scheme. Furthermore, extensive genome editing of this strain by MuGENT-SSR identified a phase-variable gene that determines the Penner serotype of NCTC11168. Thus, MuGENT-SSR provides a platform for genetic and phenotypic engineering of genetically unstable C. jejuni, making it a reliable approach for elucidating the mechanisms underlying phase-variable expression of specific phenotypes.

## INTRODUCTION

Repetitive DNA sequences are a widespread and abundant feature of genomic DNA. DNA repeats consisting of ∼1 to 6 bp in their unit structure are called “simple sequence repeats (SSRs)” (or simple DNA repeats), due to their sequence being less complex than random sequences (1, 2). The SSRs are highly mutable because of the potential for DNA strand slippage during DNA replication (3). Mispairing DNA by slippage results in the insertion or deletion of one or more repeats within a repetitive DNA element. When an SSR tract is present within an open reading frame (ORF), promoter, or other regulatory sequences, its hypermutability mediates reversible and frequent changes in specific phenotypes through transcriptional or translational genetic switches between ON and OFF states, which is called “phase variation” (4–6). In several bacterial species, including Campylobacter jejuni, the presence of multiple SSR-mediated phase-variable genes per genome generates high levels of phenotypic variants within bacterial populations (7, 8).

C. jejuni is the leading bacterial cause of foodborne gastroenteritis in developed countries, primarily because of its ability to colonize the ceca of chickens and to survive in the food chain by attaching to undercooked chicken meat (9, 10). C. jejuni readily colonizes the gastrointestinal tracts of a wide variety of wild and domestic birds and other animals (11, 12). Infections in poultry are usually asymptomatic, while human infection can cause significant inflammation, fever, headache, chill, abdominal pain, nausea, and diarrhea (10), occasionally progressing to the autoimmune neuropathies, including Guillain-Barré syndrome (GBS) (13–15). C. jejuni is also implicated in the development of irritable bowel disease (16). Disease phenotypes, including diarrhea, are less severe in developing countries than in developed countries (17, 18). In addition, in developing countries, campylobacteriosis is more frequent among children than adults, suggesting that immunity to Campylobacter might be naturally acquired as children become adults.

A relatively large number of SSR tracts consisting of seven or more G or C bases was unexpectedly found in the A/T-rich genomes of C. jejuni (8, 19). These homopolymeric polyG/C SSR tracts are involved mainly in phase variation in this species, and a genomic analysis of four C. jejuni strains indicated the presence of 12 to 29 tracts per genome (19). C. jejuni NCTC11168 encodes 29 polyG/C tracts, of which 23 are in protein-coding regions (20), suggesting that phase variation in this strain is regulated mainly at the translational level. The majority of these loci are clustered on the genomic regions predicted to encode enzymes involved in modifying surface structures, including lipooligosaccharide (LOS), capsular polysaccharide (CPS), and flagella, but a few are located on genes encoding cell surface proteins or restriction enzymes (8). In addition, homopolymeric polyA/T and heteropolymeric polyACCTT tracts, quantitatively minor SSRs in the Campylobacter genomes (21), also contribute to phase variation of the genes involved in flagellar biosynthesis and/or cell morphology (22–24). C. jejuni needs to rapidly adapt to differences in host environments, such as changing nutrient compositions and immune systems. Additional selective pressures are caused by transmission through genetically and immunologically variable host populations and exposure to bacteriophages. C. jejuni likely utilizes phase variation as a major mechanism for adapting to these selective pressures. Also, the ability to change the cell surface structures by phase variation strongly suggests that this phenotypic diversity could affect colonization or pathogenesis in host organisms. Consistently, it has been reported that phase-variable expression of specific genes affects pathogenesis in humans and colonization in chickens (22, 25–28). Comprehensive studies of multiple phase-variable genes in C. jejuni have also demonstrated that culturing or passage through animal and human hosts results in significant phase changes in multiple SSR-containing genes, particularly those involved in surface-structure modifications, which in some cases, are coupled with enhanced colonization and pathogenesis (19, 29–33).

**FIG 1:**
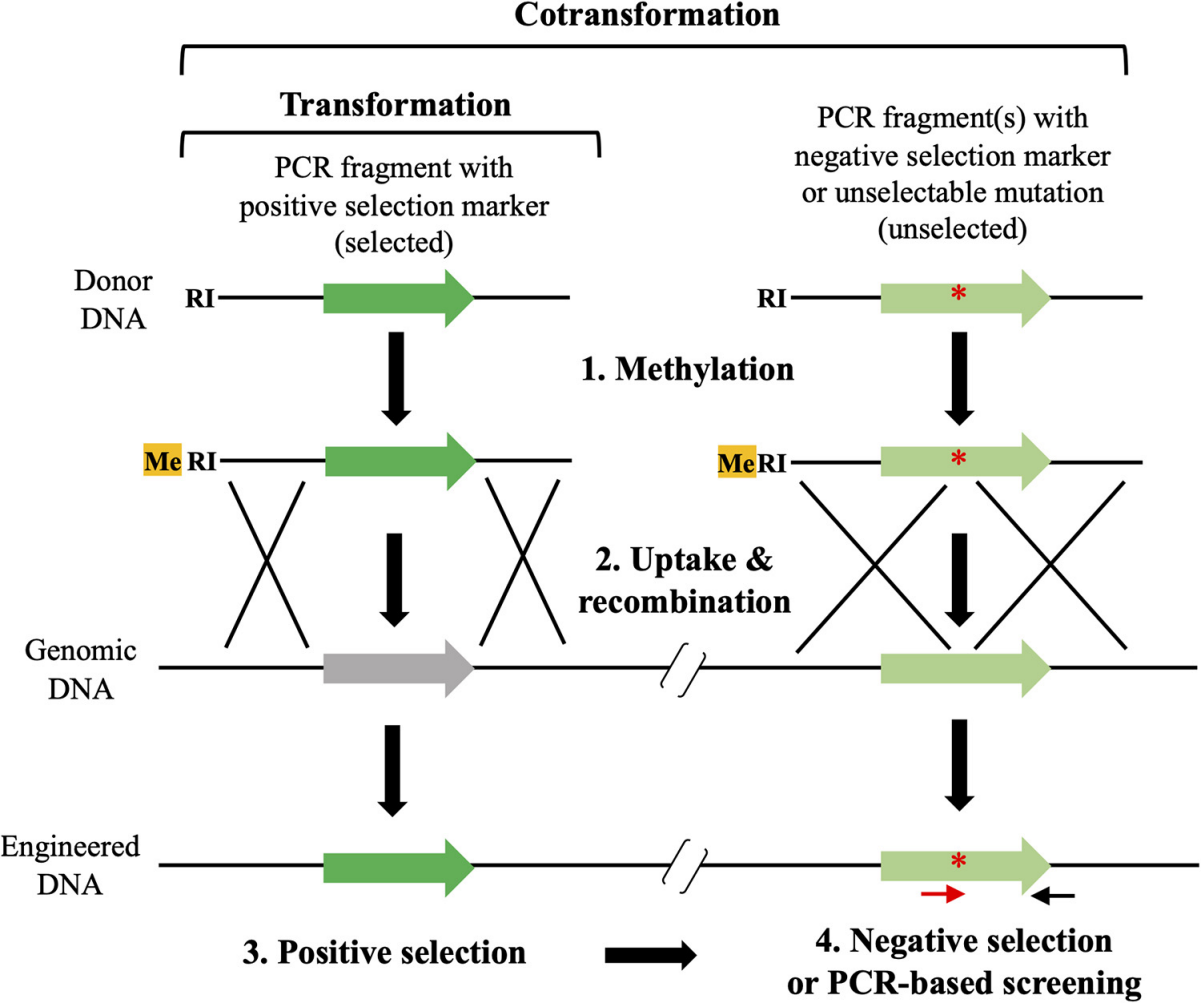
Overview of the procedures of natural transformation and cotransformation in C. jejuni in this study. First, donor DNA fragments used for natural transformation were amplified by PCR. For the C. jejuni transformation, each of the resulting PCR fragments had not only a positive selection marker (e.g., antibiotic resistance gene and gain-of-function mutation) flanking regions homologous to the target gene but also an EcoRI methyltransferase-recognition sequence, GAATTC (RI) (termed “selected” fragment). Methylated RI is thought to act as a Campylobacter-specific DNA uptake signal (39). Therefore. the next steps were to methylate the selected fragment by EcoRI methyltransferase and to use it as the substrate for DNA uptake during transformation. Finally, resulting transformants were positively selected under appropriate conditions. On the other hand, natural “cotransformation” involved the “unselected” fragment(s) that had a negative selection marker (e.g., loss-of-function mutation) or unselectable mutation (e.g., silent mutation), in addition to the selected fragment. In this case, the selected fragment was used to replace a neutral gene with a positive selection marker, while the unselected fragments were used to introduce unmarked mutations of interest at single or multiple loci. These different DNA fragments were methylated and then mixed with a recipient strain to simultaneously introduce different mutations into the genome. The cotransformation reactions were subjected to positive selection for integration of the selected fragment and subsequently screened to assess the integration of the unselected fragments by negative selection or PCR using allele-specific primers. Cotransformation has been recently adapted to multiplex genome editing by natural transformation (MuGENT), a method for introducing multiple mutations into the genomes of Vibrio cholerae and Streptococcus pneumoniae (40). In this study, we optimized natural transformation and cotransformation and demonstrated its utility as a method for multiplex genome editing in C. jejuni.

A limitation of studying the contributions of multiple SSR-mediated phase-variable genes to specific phenotypes is that researchers currently lack genetic tools for efficiently “locking” SSRs into either ON or OFF states at multiple loci in the C. jejuni genome. For example, the use of a multiplexed SSR-editing technology enables phenotype stabilization and therefore promotes reproducible research activities in this area. Such phenotypic engineering would also be applicable for stably producing phase-variable surface antigenic determinants, which can be used for vaccine development and raising serotyping antisera. To date, multiplex automated genome engineering (MAGE) using oligonucleotides and a highly efficient λRed recombinase and clustered regularly interspaced short palindromic repeats (CRISPR)-CRISPR-associated protein (Cas) systems have been developed for targeted genome engineering in bacteria (34, 35). However, these methods require not only engineered plasmids or chromosomes for the respective genome-editing systems (i.e., expressing proteins and/or RNAs involved in the λRed and CRISPR/Cas systems) but also, in the case of CRISPR-Cas, the selection of edited genomic sites. Natural competence for genetic transformation is shared by diverse bacterial species (36, 37). This process involves the uptake of exogenous DNA, followed by integration of the imported DNA into the genome by homologous recombination. Because natural transformation does not require any special factors and is dependent on the recipient bacterium and donor DNA molecules, it has long been used for genetic engineering in naturally competent bacterial species. C. jejuni was shown to be naturally transformable over 30 years ago (38). However, natural transformation has not been used conventionally as a genetic engineering method in this bacterium because it can be highly transformed with DNA prepared from C. jejuni but not with DNA propagated in Escherichia coli or amplified by the PCR. Recently, Beauchamp et al. demonstrated that methylation at the RAATTY sequence of E. coli-derived plasmid DNA or PCR-amplified DNA can efficiently transform C. jejuni (39) (Fig. 1). In a study of other transformable species conducted by Dalia et al., the authors established the multiplex genome editing by natural transformation (MuGENT) system (40), which is based on “cotransformation,” a trait that causes the uptake and integration of multiple different DNA molecules (41, 42) (Fig. 1). These two pioneering studies have provided great potential for performing multiplexed gene modifications in C. jejuni and motivated us to examine this feasibility. Here, we optimized natural transformation and cotransformation using PCR-amplified donor DNA fragments and demonstrated its utility as a method for multiplex genome editing in naturally competent C. jejuni.

## RESULTS AND DISCUSSION

### Optimization of donor DNA for the natural transformation of C. jejuni.

**FIG 2:**
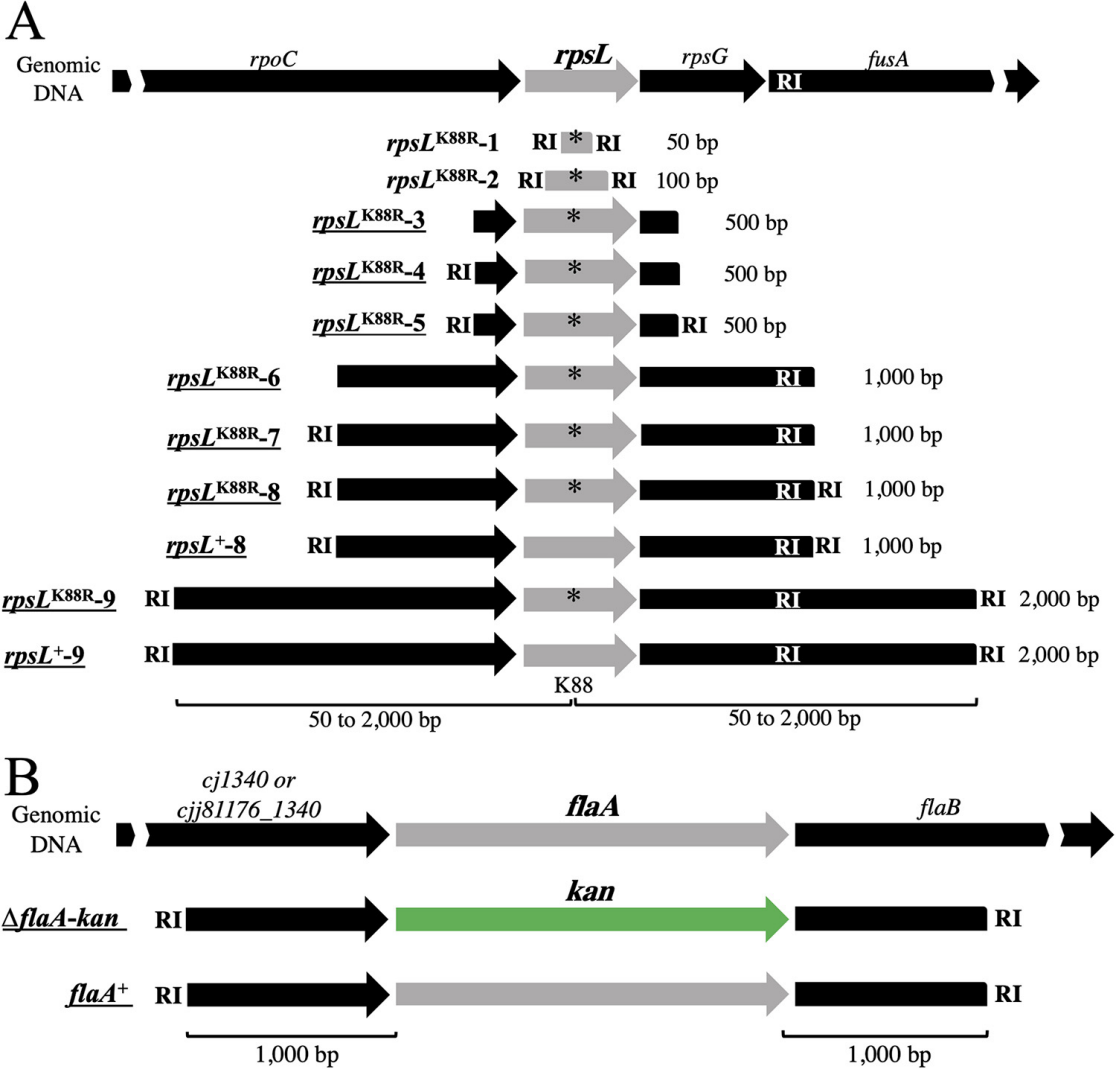
Schematic representation of donor DNA fragments used for the optimization of natural transformation and cotransformation in the recipient C. jejuni strains NCTC11168 and 81-176. (A) *rpsL* fragments with different lengths of homologous regions compared with the recombination target sequence (50 to 2,000 bp, indicated in the right) and different numbers of EcoRI methyltransferase-recognition sequences (indicated by RIs). An asterisk indicates the *rpsL*^K88R^ mutation that confers streptomycin resistance. (B) *flaA* fragments with 1,000-bp homologous regions and two RIs. The Δ*flaA*::*kan* was generated by replacing *flaA* with a kanamycin resistance gene (*kan*), whereas the *flaA*^+^ contained an intact *flaA* sequence. In both panels, each of the fragments with underlines (indicated in the left, *rpsL*^K88R^-3 to -9, *rpsL*^+^-8, *rpsL*^+^-9, Δ*flaA*::*kan*, and *flaA*^+^) consisted of two sequence variants to be used for their respective recipient strains (see Table S2, fragments labeled “A” for NCTC11168 and labeled “B” for 81-176, e.g., *rpsL*^K88R^-3 consisted of *rpsL*^K88R^-3-A and *rpsL*^K88R^-3-B for NCTC11168 and 81-176, respectively) because of genomic variations at the target sequences in these strains. Sequences of the fragments not underlined (*rpsL*^K88R^-1 and *rpsL*^K88R^-2) were identical between NCTC11168 and 81-176 and thus were shared with these strains.

Data from a previous study by Beauchamp et al. demonstrated that DNA methylated at the RA^m6^ATTY sequence is an efficient substrate for C. jejuni transformation (39) (Fig. 1). To optimize the natural transformation of this bacterium, we investigated in greater detail the characteristics of DNA substrate generated using PCR products. For this purpose, we amplified various DNA fragments of the *rpsL*^K88R^ allele, which confers streptomycin resistance (Sm^r^) (Fig. 2A) (43). These *rpsL*^K88R^ marker fragments had different lengths of regions homologous to the recombination target sequence (50 to 2,000 bp) and different numbers of the EcoRI methyltransferase-recognition sequence (GAATTC), which was present endogenously or added exogenously to the PCR primers. Each 0.5 pmol of the amplified DNA was treated with EcoRI methyltransferase and then used in transformation assays to transform the NCTC11168 and 81-176 strains, which are representative strains used for genetic studies of C. jejuni (8, 44, 45) (Table 1). We did not obtain transformants using fragments with short regions of homology (50 or 100 bp), even though these DNA fragments had two methylated GAATTC sites (Table 2, see *rpsL*^K88R^-1 and *rpsL*^K88R^-2). In contrast, the NCTC11168 and 81-176 strains were transformed at substantially higher frequencies using fragments with longer regions of homology (500 bp) (Table 2, see *rpsL*^K88R^-5). The highest transformation frequencies were obtained when 1,000- to 2,000-bp homologies were present (Table 2, see *rpsL*^K88R^-8 and *rpsL*^K88R^-9). The length of a homologous sequence is a major determinant of the efficiency of RecA-dependent recombination (46). In the Δ*recA* background, no transformants were detected using a fragment with 2,000 homologous bp (Table 2, see *rpsL*^K88R^-9), suggesting that transformation in C. jejuni is mediated by RecA. We also confirmed that the lack of the GAATTC sequence or the lack of GAATTC methylation markedly decreased the transformation frequency (Table 2, see *rpsL*^K88R^-3 and unmethylated *rpsL*^K88R^-5). However, increasing the number of methylated GAATTC sites did not substantially increase the transformation frequencies in NCTC11168 (Table 2, compare *rpsL*^K88R^-4 and *rpsL*^K88R^-5 or *rpsL*^K88R^-6, *rpsL*^K88R^-7, and *rpsL*^K88R^-8). In 81-176, increasing the number of these sites from 1 to 2 increased the transformation frequency by approximately 30-fold (Table 2, compare *rpsL*^K88R^-6 and *rpsL*^K88R^-7), but increasing from 2 to 3 did not (Table 2, compare *rpsL*^K88R^-7 and *rpsL*^K88R^-8). These results suggest that one methylated GAATTC sequence is sufficient for transformation, consistent with previous results obtained using plasmid DNA as a donor (39). In summary, to maximize the efficiency of C. jejuni transformation, the donor DNA should contain the following two key structural elements: (i) a ≥1,000-bp region of homology and (ii) at least one methylated GAATTC site.

**TABLE 1:** Strains and plasmids used in this study

Strain or plasmid	Characteristics	Source or reference
Strain		
NCTC11168	C. jejuni human origin, serotype B (antigenic factor HS2)	44
SYC1001	NCTC11168 Δ*flaA*::*cat*	This study
SYC1002	NCTC11168 Δ*flaA*::*kan*	This study
SYC1003	NCTC11168 *rpsL*^K88R^	This study
SYC1004	NCTC11168 Δ*recA*::*cat*	This study
SYC1006	NCTC11168 *cj1426*::*astA* Δ*flaA*::*kan*	This study
SYC1007	NCTC11168 *cj1426*^ON^::*astA* Δ*flaA*::*cat*	This study
SYC1008	NCTC11168 *cj1426*^OFF^::*astA* Δ*flaA*::*kan*	This study
SYC1P000K	NCTC11168 Δ*flaA*::*kan cj1139*^OFF^ *cj1144*^OFF^ *cj1420*^OFF^ *cj1421*^OFF^ *cj1422*^OFF^ *cj1426*^OFF^ *cj1429*^OFF^ *cj1437*^OFF^	This study
SYC1P000	NCTC11168 *cj1139*^OFF^ *cj1144*^OFF^ *cj1420*^OFF^ *cj1421*^OFF^ *cj1422*^OFF^ *cj1426*^OFF^ *cj1429*^OFF^ *cj1437*^OFF^	This study
SYC1P001C	NCTC11168 Δ*flaA*::*cat cj1139*^OFF^ *cj1144*^OFF^ *cj1420*^OFF^ *cj1421*^OFF^ *cj1422*^OFF^ *cj1426*^OFF^ *cj1429*^OFF^ *cj1437*^ON^	This study
SYC1P004K	NCTC11168 Δ*flaA*::*kan cj1139*^OFF^ *cj1144*^OFF^ *cj1420*^OFF^ *cj1421*^OFF^ *cj1422*^OFF^ *cj1426*^ON^ *cj1429*^OFF^ *cj1437*^OFF^	This study
SYC1P005C	NCTC11168 Δ*flaA*::*cat cj1139*^OFF^ *cj1144*^OFF^ *cj1420*^OFF^ *cj1421*^OFF^ *cj1422*^OFF^ *cj1426*^ON^ *cj1429*^OFF^ *cj1437*^ON^	This study
SYC1P032K	NCTC11168 Δ*flaA*::*kan cj1139*^OFF^ *cj1144*^OFF^ *cj1420*^ON^ *cj1421*^OFF^ *cj1422*^OFF^ *cj1426*^OFF^ *cj1429*^OFF^ *cj1437*^OFF^	This study
SYC1P033C	NCTC11168 Δ*flaA*::*cat cj1139*^OFF^ *cj1144*^OFF^ *cj1420*^ON^ *cj1421*^OFF^ *cj1422*^OFF^ *cj1426*^OFF^ *cj1429*^OFF^ *cj1437*^ON^	This study
SYC1P036C	NCTC11168 Δ*flaA*::*cat cj1139*^OFF^ *cj1144*^OFF^ *cj1420*^ON^ *cj1421*^OFF^ *cj1422*^OFF^ *cj1426*^ON^ *cj1429*^OFF^ *cj1437*^OFF^	This study
SYC1P037C	NCTC11168 Δ*flaA*::*cat cj1139*^OFF^ *cj1144*^OFF^ *cj1420*^ON^ *cj1421*^OFF^ *cj1422*^OFF^ *cj1426*^ON^ *cj1429*^OFF^ *cj1437*^ON^	This study
SYC1P037	NCTC11168 *cj1139*^OFF^ *cj1144*^OFF^ *cj1420*^ON^ *cj1421*^OFF^ *cj1422*^OFF^ *cj1426*^ON^ *cj1429*^OFF^ *cj1437*^ON^	This study
SYC1P255K	NCTC11168 Δ*flaA*::*kan cj1139*^ON^ *cj1144*^ON^ *cj1420*^ON^ *cj1421*^ON^ *cj1422*^ON^ *cj1426*^ON^ *cj1429*^ON^ *cj1437*^ON^	This study
SYC1P255	NCTC11168 *cj1139*^ON^ *cj1144*^ON^ *cj1420*^ON^ *cj1421*^ON^ *cj1422*^ON^ *cj1426*^ON^ *cj1429*^ON^ *cj1437*^ON^	This study
81-176	C. jejuni raw milk origin, serotype R (antigenic factors HS23/HS36)	45
SYC2001	81-176 Δ*flaA*::*cat*	This study
SYC2002	81-176 Δ*flaA*::*kan*	This study
SYC2003	81-176 *rpsL*^K88R^	This study
SYC2004	81-176 Δ*recA*::*cat*	This study
Plasmid		
pUCFa	Cloning vector, *bla*	Fasmac
pSYC-*cat*	pUCFa *cat* from Campylobacter coli	This study
pSYC-*kan*	pUCFa *kan* from C. coli	This study

**TABLE 2:** Effects of homology length and methylation on natural transformation

Recipient strain^*a*^	Donor DNA^*b*^	Homology length (bp)	No. of GAATTC sites	Methylation reaction^*c*^	Transformation frequency^*d*^
NCTC11168 WT	*rpsL*^K88R^-1	50	2	+	<1.5 × 10^−8^
	*rpsL*^K88R^-2	100	2	+	<1.4 × 10^−8^
	*rpsL*^K88R^-3	500	0	+	1.8 ± 2 × 10^−8^
	*rpsL*^K88R^-4	500	1	+	1.2 ± 1 × 10^−6^
	*rpsL*^K88R^-5	500	2	+	3.2 ± 3 × 10^−6^
	*rpsL*^K88R^-5	500	2	−	2.7 ± 1 × 10^−8^
	*rpsL*^K88R^-6	1,000	1	+	1.2 ± 1 × 10^−5^
	*rpsL*^K88R^-7	1,000	2	+	9.0 ± 1 × 10^−6^
	*rpsL*^K88R^-8	1,000	3	+	2.0 ± 1 × 10^−5^
	*rpsL*^K88R^-9	2,000	3	+	5.0 ± 2 × 10^−5^
	-	Not applicable	Not applicable	Not applicable	<1.2 × 10^−8^
NCTC11168 Δ*recA*	*rpsL*^K88R^-9	2,000	3	+	<1.6 × 10^−7^
81-176 WT	*rpsL*^K88R^-1	50	2	+	<2.1 × 10^−8^
	*rpsL*^K88R^-2	100	2	+	<2.2 × 10^−8^
	*rpsL*^K88R^-3	500	0	+	1.4 ± 1 × 10^−8^
	*rpsL*^K88R^-4	500	1	+	2.1 ± 2 × 10^−5^
	*rpsL*^K88R^-5	500	2	+	3.1 ± 1 × 10^−5^
	*rpsL*^K88R^-5	500	2	−	1.2 ± 3 × 10^−8^
	*rpsL*^K88R^-6	1,000	1	+	4.0 ± 1 × 10^−5^
	*rpsL*^K88R^-7	1,000	2	+	1.3 ± 1 × 10^−4^
	*rpsL*^K88R^-8	1,000	3	+	1.3 ± 1 × 10^−4^
	*rpsL*^K88R^-9	2,000	3	+	1.2 ± 1 × 10^−4^
	-	Not applicable	Not applicable	Not applicable	<1.9 × 10^−8^
81-176 Δ*recA*	*rpsL*^K88R^-9	2,000	3	+	<1.4 × 10^−7^

a The following strains were used: NCTC11168 WT, NCTC11168; NCTC11168 Δ*recA*, SYC1004; 81-176 WT, 81-176; 81-176 Δ*recA*, and SYC2004.

b The donor DNA fragments are shown in Fig. 2; -, indicates no addition of DNA.

c Each 0.5 pmol of DNA was treated (+) or not treated (−) witd EcoRI metdyltransferase and tden used in tde transformation assays.

d The transformation frequency was defined as tde number of streptomycin-resistant CFU divided by tde total number of CFU. Data from tdree independent transformations are presented as tde avg ± SD.

10.1128/mBio.01401-21.6 TABLE S2 Specific combinations of template DNA and primers used to amplify donor DNA fragments. Download Table S2, PDF file, 0.1 MB. Copyright © 2021 Yamamoto et al. 2021 Yamamoto et al. https://creativecommons.org/licenses/by/4.0/ This content is distributed under the terms of the Creative Commons Attribution 4.0 International license.

### Occurrence of natural cotransformation in C. jejuni.

Using Vibrio cholerae and Streptococcus pneumoniae, Dalia et al. developed the MuGENT system using cotransformation (40), a trait of several naturally competent species (41, 42). During MuGENT, a bacterial culture is incubated with the following two types of donor DNA fragments: (i) a selected fragment that introduces an antibiotic resistance gene into the genome and (ii) unselected fragments that introduce scarless or transgene-free edits of interest at one or more loci (Fig. 1). In V. cholerae, the frequencies of cotransformation of these distinct genetic markers can be made to exceed 60% by increasing the length of homology and the concentration of the unselected fragment (40). To assess natural cotransformation in C. jejuni, we used two strains (SYC1003 and SYC2003) as recipients, which harbor the *rpsL*^K88R^ mutations in the NCTC11168 and 81-176 genomes, respectively, and therefore are resistant to Sm. We also used a Δ*flaA*::*kan* fragment with 1,000-bp regions of homology to replace the flagellin gene with a kanamycin resistance (Km^r^) marker (selected) (Fig. 2B) and *rpsL*^+^ fragments with homologous regions of different sizes (1,000 bp, *rpsL*^+^-8; 2,000 bp, *rpsL*^+^-9) (Fig. 2A) to revert to the wild-type Sm-sensitive (Sm^s^) phenotype (unselected). After transforming SYC1003 and SYC2003 with equimolar concentrations of the selected and unselected methylated DNA (mDNA) fragments, we selected Km^r^ transformants, of which 100 were then subjected to Sm-sensitivity testing to evaluate cotransformation (Table 3). Using unselected mDNA with a 1,000-bp region of homology resulted in cotransformation frequencies of 26% and 13% in SYC1003 and SYC2003, respectively, and acquisition of the Km^r^ and Sm^s^ phenotypes (Table 3). Furthermore, increasing the homology of unselected mDNA (2,000 bp) increased the cotransformation frequencies of both strains (Table 3) (1.8- and 1.9-fold increases in SYC1003 and SYC2003, respectively). Thus, we currently recommend the use of unselected mDNA with 2,000-bp homology for efficient C. jejuni transformation. However, increasing the concentration of the unselected fragment (3-fold increase) increased the cotransformation frequency of SYC1003 (Table 3, 1.8-fold increase) but not SYC2003, suggesting that there may be strain-dependent concentration effects.

**TABLE 3:** Evaluation of natural C. jejuni cotransformation

Recipient strain	Donor DNA^*a*^		Cotransformation frequency^*b*^
	Selected	Unselected	
SYC1003	Δ*flaA*::*kan* (0.5)	*rpsL*^+^-8 (0.5)	26 ± 7
		*rpsL*^+^-9 (0.5)	46 ± 11
		*rpsL*^+^-9 (1.5)	82 ± 8
SYC2003	Δ*flaA*::*kan* (0.5)	*rpsL*^+^-8 (0.5)	13 ± 4
		*rpsL*^+^-9 (0.5)	25 ± 5
		*rpsL*^+^-9 (1.5)	24 ± 3

a The selected and unselected DNA fragments are shown in Fig. 2. Value in parentheses indicates the amt of DNA used for methylation (pmol).

b The cotransformation frequency (%) was calculated as follows: 100 × number of streptomycin-sensitive CFU per 100 kanamycin-resistant CFU. Data from six independent transformations are presented as the avg ± SD.

Natural cotransformation is thought to reflect the nature of competent bacterial cells, among which only a subpopulation of cells in a culture is transformable (41). We demonstrated for the first time that C. jejuni cells are capable of cotransformation. It will be important to elucidate whether C. jejuni cells show variable competence within a population.

### Establishment of MuGENT in C. jejuni.

**FIG 3:**
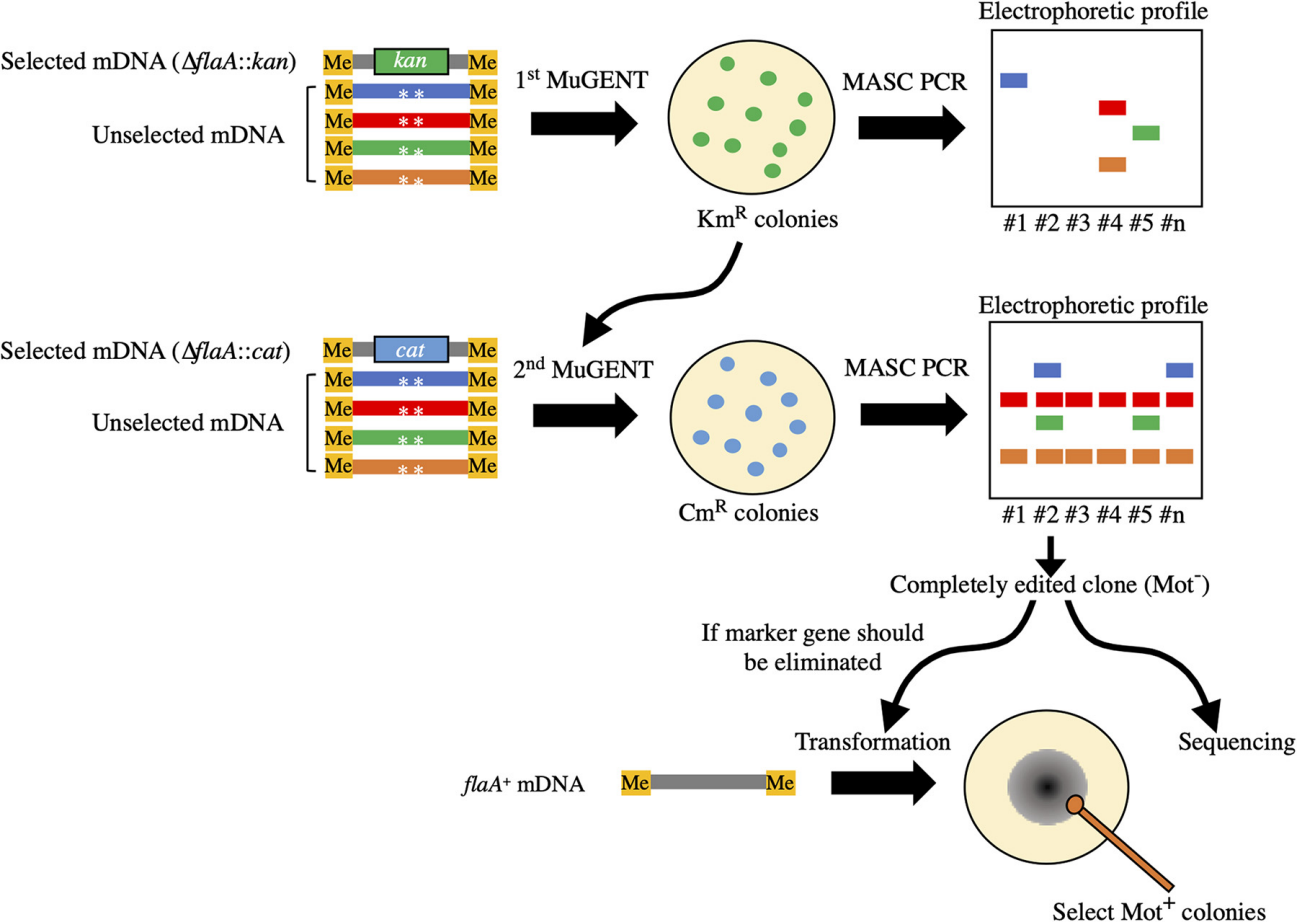
Overview of MuGENT in C. jejuni. Multiple unselected mDNA fragments (used for introducing genome edits of interest) were mixed with a selected mDNA fragment in order to cotransform C. jejuni cells. During MuGENT, we swapped different selective markers at the *flaA* gene during every cycle, resulting in the cells becoming nonmotile (Mot^−^). This also enabled easy removal of marker genes and reversion to the wild-type allele by transformation with a *flaA*^+^ mDNA and subsequent selection of motile (Mot^+^) cells (see Materials and Methods). Genome edits in the transformants were verified by MASC PCR and nucleotide sequencing.

MuGENT provides methods for simultaneously generating multiple scarless mutations and can therefore be broadly applied in diverse research and biotechnology applications (40). We wanted to test whether natural cotransformation could be used for multiplex genome editing of C. jejuni. Figure 3 presents a schematic representation of our strategy. Briefly, multiple unselected mDNA fragments (used for introducing genome edits of interest) were mixed with a selected mDNA fragment, and the resulting mixture was used to cotransform C. jejuni cells. Because MuGENT often requires multiple cycles of cotransformation in order to complete the genome editing, different selectable markers should be used for each cycle (40). We swapped different resistance markers at the *flaA* gene at every MuGENT cycle. This procedure also enabled the easy removal of the marker genes and reversion to the wild-type allele by transformation with *flaA*^+^ mDNA (Fig. 2B) and subsequent selection of motile clones. Genome editing was verified by multiplex allele-specific colony (MASC) PCR (47) and nucleotide sequencing.

To demonstrate the efficacy of MuGENT in C. jejuni, we targeted the biosynthetic genes CPS and LOS. These cell surface molecules are known to play key roles in interactions that affect bacteriophage infectivity, chick colonization, invasion of human epithelial cells, and host immune responses (34–40). CPS is also the primary antigenic determinant of the Penner serotyping scheme (48), a passive slide hemagglutination, although other surface molecules, including LOS, may contribute to serotype specificity (49). Because the CPS and LOS gene clusters contain multiple phase-variable genes that are interrupted by polyG tracts, C. jejuni cells can generate structural variations of CPS and LOS, which aids in evading killing by host immune systems or predation by bacteriophages (26, 50–55). In addition, phase-variable expression of CPS and LOS markedly decreases the phenotypic stability of C. jejuni cells and, thus, may limit related research, including basic studies, epidemiological surveillance, and vaccine development. For example, the NCTC11168 strain (serotype B, antigenic factor HS2) did not provide reproducible results for Penner serotyping, which frequently changed between typeable and untypeable during subcultures (see Fig. S1 in the supplemental material). Although this unstable phenotype may be attributable to ON/OFF switching of the CPS expression due to phase variation (48, 56, 57), the mechanism whereby phase variation regulates CPS gene expression to determine the Penner serotype remains unknown.

10.1128/mBio.01401-21.1 FIG S1 Penner serotyping of naturally occurring and phase-locked NCTC11168 variants. Phenotypic stability was examined during successive subcultures, which highlights only the results obtained using antiserotype B antiserum. Red-numbered colonies were typeable, and black-numbered colonies were untypeable. Download FIG S1, PDF file, 0.2 MB. Copyright © 2021 Yamamoto et al. 2021 Yamamoto et al. https://creativecommons.org/licenses/by/4.0/ This content is distributed under the terms of the Creative Commons Attribution 4.0 International license.

**FIG 4:**
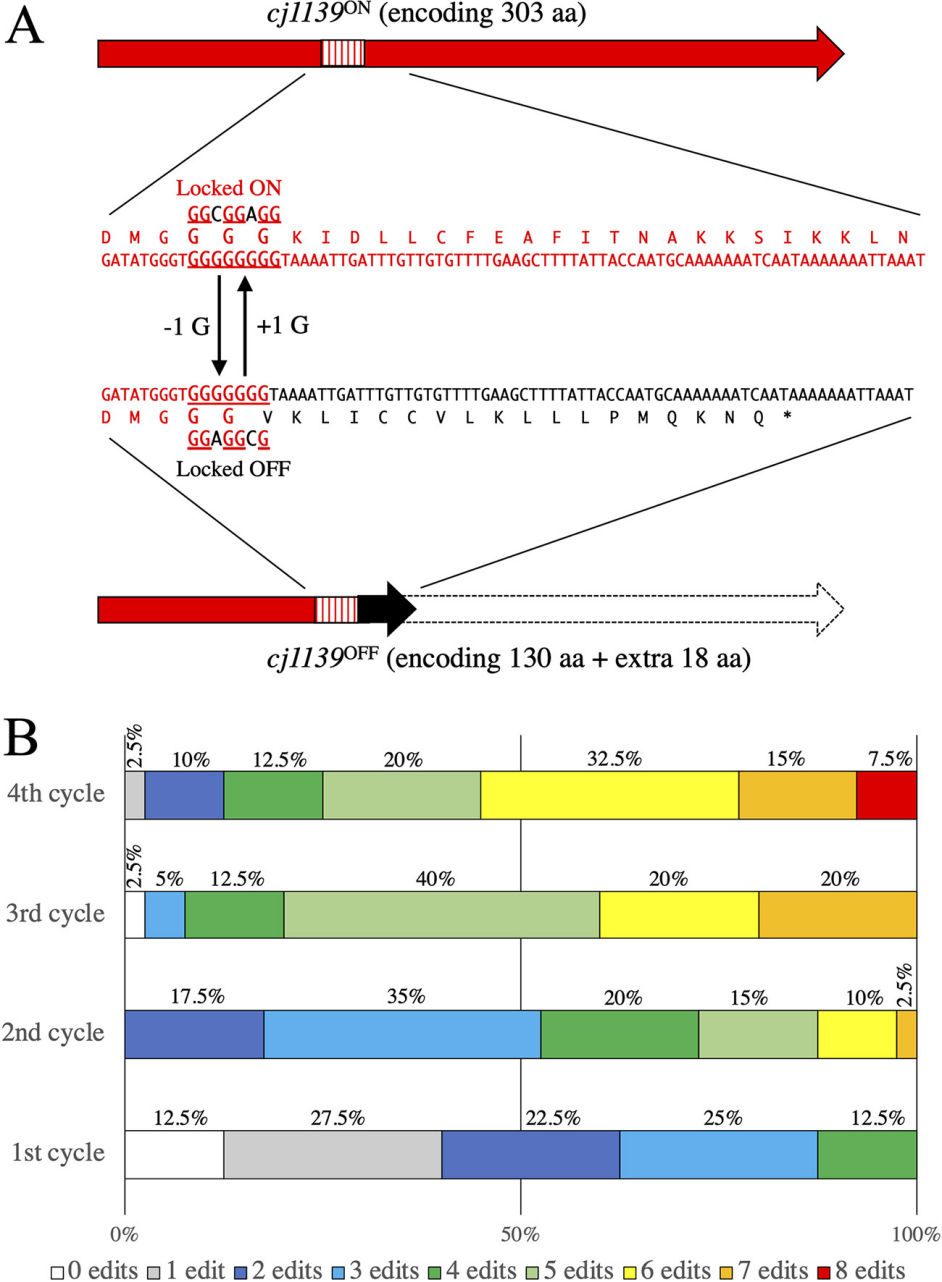
Constructing a strain with all eight phase-variable genes locked into the OFF state by MuGENT against SSR (MuGENT-SSR) sites. The polyG tracts from eight genes (*cj1139*, *cj1144*, *cj1420*, *cj1421*, *cj1422*, *cj1426*, *cj1429*, and *cj1437*) located in the biosynthetic gene clusters for LOS and CPS in NCTC11168 were subjected to MuGENT-SSR to lock them all into OFF states. (A) Example of polyG editing (*cj1139*). The polyG tract was altered to translate into the largest possible ORF (G8, ON state, encoding 303 amino acids [aa]) or a smaller incomplete ORF by introducing frameshifts via −1 deletions (G7, OFF state, encoding 148 amino acids). To prevent replicative slippage at the polyG tract, we interrupted the continuous run of G residues without changing the translated amino acids by replacing the last G residue of every G triplet with a different nucleotide. We also used different nucleotides when locking the genes into ON and OFF states, so that these two different states could be swapped. (B) Distribution of the numbers of genome edits (0 to 8 edits) in the population following successive cycles of MuGENT. This image was based on the MASC PCR data of 40 random mutants per cycle (Fig. S2).

There are 29 polyG/C tracts in the NCTC11168 genome, of which 8 are located within the CPS and LOS gene clusters (20). Six of these phase-variable genes (*cj1420*, *cj1421*, *cj1422*, *cj1426*, *cj1429*, and *cj1437*) reside in the CPS cluster, whereas the other genes (*cj1139* and *cj1144*) reside in the LOS cluster, which theoretically gives rise to ∼2^8^ different combinations of ON (“1”)/OFF (“0”) expression states or more specifically “phasotypes” (7). For example, a bacterium that has the expression states *cj1139*^ON^ *cj1144*^OFF^ *cj1420*^OFF^ *cj1421*^OFF^ *cj1422*^OFF^ *cj1426*^OFF^ *cj1429*^OFF^ *cj1437* ^OFF^ (in that order) would have a binary phasotype coded as 1-0-0-0-0-0-0-0, which can be converted to a decimal 128 format. In this study, we defined one of the 2^8^ phasotypes generated by these eight phase-variable genes as a “Penner phasotype (PPT).” Using MuGENT, we tried to “lock” all of the eight ORFs into ON or OFF states, where their polyG tracts were altered to translate into the largest possible ORF (locked-ON states) or a smaller incomplete ORF by frameshifting through −1 deletions from the ON states (locked-OFF states). For example, *cj1139* (*wlaN*) encodes a glucosyltransferase that mediates phase variation of LOS epitopes responsible for autoimmunity in GBS (27), with G8 being in an ON state and G7 in an OFF state (Fig. 4A). To prevent replicative slippage at the polyG tracts, we interrupted the continuous run of G residues without changing the translated amino acids by replacing the last G residue of every G triplet with a different nucleotide (Fig. 4A). As an example of phase-locked mutant construction, we introduced locked-OFF mutations into *cj1139*, *cj1144*, *cj1420*, *cj1421*, *cj1422*, *cj1426*, *cj1429*, and *cj1437* by repeating the MuGENT cycle to generate a “PPT0” strain. During MuGENT, genome editing from 40 transformants per cycle was monitored by MASC PCR (see Fig. S2 in the supplemental material). After the second cycle, we found that 100% of the population had at least one edit (Fig. 4B and Fig. S2). We accomplished all eight edits within the 4th cycle (Fig. 4B and Fig. S2), which took less than 2 weeks to perform. Thus, MuGENT works effectively in C. jejuni and is useful for rapidly introducing multiple mutations.

10.1128/mBio.01401-21.2 FIG S2 MASC PCR of transformants following successive cycles of MuGENT-SSR. Forty transformants per cycle were subjected to MASC PCR using the primer mixes mix OFF1 and mix OFF2 (Table S4). Edit number indicates the number of genome edits of each transformant. Transformants with all eight genome edits are shown in red. Download FIG S2, PDF file, 0.6 MB. Copyright © 2021 Yamamoto et al. 2021 Yamamoto et al. https://creativecommons.org/licenses/by/4.0/ This content is distributed under the terms of the Creative Commons Attribution 4.0 International license.

### Improving the phenotypic stability during Penner serotyping using MuGENT.

**FIG 5:**
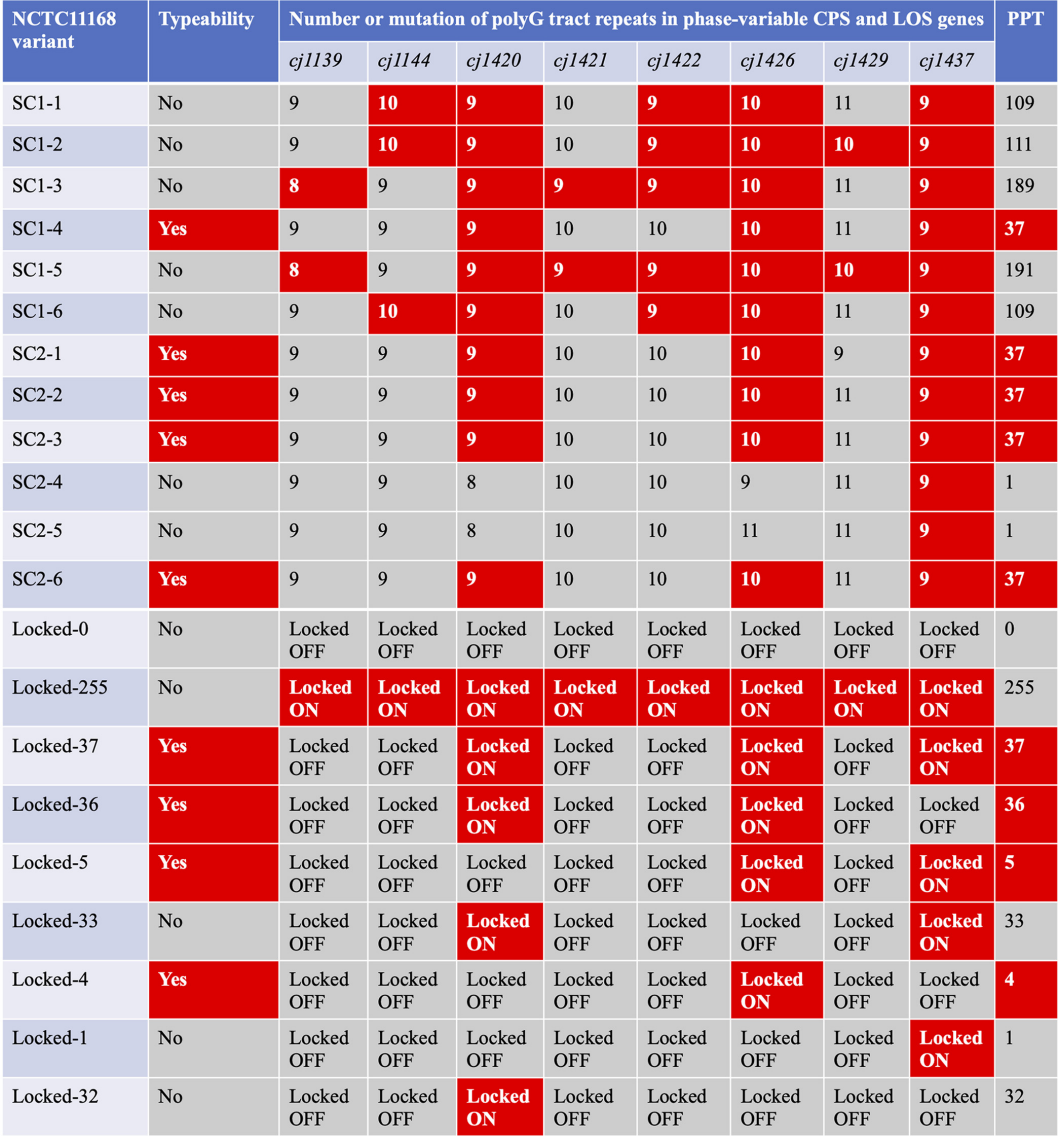
Penner serotyping and phasotyping of naturally occurring and phase-locked variants of NCTC11168. After naturally occurring variants (typeable or untypeable by Penner serotyping; SC1-1 to SC2-6) (Fig. S1) were subjected to PPT-Seq of the eight phase-variable CPS and LOS genes (*cj1139*, *cj1144*, *cj1420*, *cj1421*, *cj1422*, *cj1426*, *cj1429*, and *cj1437*), their PPTs were decoded (indicated by decimal in the right column). In the naturally occurring variants, the numbers within red boxes indicate the numbers of repeat polyG tracts in the ON configurations, whereas those in the gray boxes indicate those in the OFF configurations. In the phase-locked variants (locked 0, locked-255, locked-37, locked-36, locked-5, locked-33, locked-4, locked-1, and locked-32), the locked-ON and locked-OFF mutations introduced by MuGENT-SSR are indicated in the red and gray boxes, respectively. The serotyping results for the phase-locked variants are shown in Fig. S3. The following phase-locked strains were used: locked-0, SYC1P000; locked-255, SYC1P255; locked-37, SYC1P037; locked-36, SYC1P036C; locked-5, SYC1P005C; locked-33, SYC1P033C; locked-4, SYC1P004K; locked-1, SYC1P001C; locked-32, SYC1P032K.

Controlling SSR-mediated phase variation is important for achieving reproducible results with C. jejuni. Regarding the low reproducibility of Penner serotyping (Fig. S1), we hypothesized that it may be associated with the phase-variable expression of one or more genes that determine antigenicity. To narrow down candidate genes, we sequenced PPT from naturally occurring typeable and untypeable variants of NCTC11168, termed “PPT-Seq.” PPT-Seq analysis of 12 variants revealed 6 PPT subtypes, of which 1 was typeable (Fig. 5 and Fig. S1) (PPT37). We constructed a strain locked to PPT37 by MuGENT and confirmed that it was also typeable (Fig. 5 and Fig. S1). In contrast to the wild-type strain, the locked PPT37 mutant maintained typeability during at least five subcultures (Fig. S1). To evaluate the effect of polyG editing by MuGENT on phase variation, the *astA* gene (encoding arylsulfatase) (58) was fused in frame to the phase-variable gene *cj1426*, which encodes a methyltransferase that methylates the heptose of the CPS repeat unit of NCTC11168 (59), and to its phase-locked derivatives, such that changes in the repeat number would alter arylsulfatase expression (60). Single colonies grown on brain heart infusion (BHI) agar plates containing 5-bromo-4-chloro-3-indolyl sulfate (XS), a chromogenic substrate of arylsulfatase, were used to measure mutation rates, as described in the Materials and Methods. With the wild-type *cj1426*::*astA* fusion, the ON-to-OFF mutation occurred at a rate of 2.8 × 10^−2^, whereas the OFF-to-ON mutation rate was 2.3 × 10^−2^ (Table 4). In the locked-ON and locked-OFF constructs, however, switching to different phases was not detectable (Table 4). These results suggest that MuGENT can serve as a feasible and effective approach for stabilizing unstable phenotypes generated by phase variation with a defined set of multiple SSRs.

**TABLE 4:** Effect of the polyG editing on phase variation of *cj1426*

Reporter gene^*a*^	Mutation rate for^*b*^:	
	ON to OFF	OFF to ON
*cj1426*::*astA*	2.8 × 10^−2^	2.3 × 10^−2^
*cj1426*^ON^::*astA*	<2.1 × 10^−3^	ND^*c*^
*cj1426*^OFF^::*astA*	ND	< 2.2 × 10^−3^

a The following strains were used: *cj1426*::*astA*, SYC1006; *cj1426*^ON^::*astA*, SYC1007; and *cj1426*°^FF^::*astA*, SYC1008.

b Mutation rates were calculated using the equation reported previously by Drake (82) (see Materials and Methods).

c ND, not done.

10.1128/mBio.01401-21.3 FIG S3 Penner serotyping of phase-locked NCTC11168 variants. See Fig. 5 for strains used. Wells serotyped as “B” are marked with red squares. Download FIG S3, PDF file, 0.2 MB. Copyright © 2021 Yamamoto et al. 2021 Yamamoto et al. https://creativecommons.org/licenses/by/4.0/ This content is distributed under the terms of the Creative Commons Attribution 4.0 International license.

### Using MuGENT-SSR to identify a phase-variable gene that determines the Penner serotype of NCTC11168.

Phenotypic engineering using MuGENT-SSR combined with PPT-Seq identified PPT37 as a PPT subtype typeable for Penner serotyping. In PPT37, *cj1420*, *cj1426*, and *cj1437* were in the ON state, whereas the other five genes were in the OFF state. These findings suggest that one or more genes of these ON genes may determine typeability. To elucidate the responsible gene(s), we constructed strains locked to various PPT subtypes by MuGENT-SSR and then performed serotyping. We demonstrated that *cj1426* expression was indispensable and sufficient for serological typing, whereas *cj1420* and *cj1437* expression were not (Fig. 5 and Fig. S3 in the supplemental material). The CPS repeat unit of C. jejuni NCTC11168 consists of β-d-ribose, β-d-*N*-acetylgalactosamine (GalfNAc), α-d-glucuronic acid modified with 2-amino-2-deoxyglycerol at C-6, and 3,6-*O*-methyl-d-glycero-a-l-gluco-heptose (Hep) (61) (see Fig. S4 in the supplemental material). Examination of the CPS structure by high-resolution magic-angle spinning nuclear magnetic resonance spectroscopy revealed highly variable or no modification of the methyl (Me), ethanolamine, aminoglycerol, and phosphoramidate groups of the repeating unit (62, 63). In particular, changes in methyl and phosphoramidate modifications were regulated by at least three phase-variable genes located in the CPS cluster (*cj1421*, *cj1422*, and *cj1426*). The *cj1426* gene encodes the methyltransferase involved in 6-O-Me modification of the Hep residue of NCTC11168 CPS (59) (Fig. S4), suggesting that the presence of this modification may be an antigenic determinant of the Penner B serotype (or antigenic factor HS2). However, we observed that some PPT subtypes (including naturally occurring variants PPT109, PPT111, PPT189, and PPT191 and a phase-locked variant, PPT255), where *cj1426* and some or all of the other phase-variable genes (including *cj1144*, *cj1421*, *cj1422*, and *cj1429*) were simultaneously switched ON, were untypeable (Fig. 5, Fig. S1 and S^3^). Of these genes, the *cj1421* and *cj1422* genes have already been demonstrated to encode *O*-Me-phosphoramidate (MeOPN) transferases that attach MeOPN to the GalfNAc and Hep residues, respectively (38). Thus, one or more CPS modifications catalyzed by these gene products may interfere with the binding of specific antibodies to the epitope (Fig. S4). To directly test this possibility, structural analysis using nuclear magnetic resonance spectroscopy and deeper genetic and immunogenic studies are required.

10.1128/mBio.01401-21.4 FIG S4 Putative modification patterns of the CPS repeat unit in naturally occurring and phase-locked NCTC11168 variants. Figures were modified from illustrations published in Sternberg et al. (59). Abbreviations for the sugars and modifications are as follows: MeOPN, *O*-methyl-phosphoramidate; NGro, *N*-glycerol; Rib, ribose; GalfNAc, *N*-acetylgalactosamine in the furanose configuration; GlcA, glucuronic acid; 6-OMe, 6-*O*-methyl; Hep, heptose, 3-OMe, 3-*O*-methyl. Download FIG S4, PDF file, 0.09 MB. Copyright © 2021 Yamamoto et al. 2021 Yamamoto et al. https://creativecommons.org/licenses/by/4.0/ This content is distributed under the terms of the Creative Commons Attribution 4.0 International license.

Similar results were reported previously regarding the phase-variable CPS of C. jejuni. In a series of studies on the tropism of Campylobacter bacteriophages, Sørensen et al. demonstrated that the MeOPN-modified GalfNAc of the NCTC11168 CPS acts as a receptor of the F336 phage but that its receptor function was modulated by the presence or absence of other CPS modifications (53, 55). In addition, a study of C. jejuni 81-176 (Penner serotype R, antigenic factors HS23 and HS36) revealed that phase-variable MeOPN modifications at the three CPS galactose residues modulated serum resistance (50). These previous findings and our current findings suggest that phase-variable changes in the CPS structure of C. jejuni occur at the following two levels: (i) phase variations of the receptors and epitopes themselves and (ii) interference with receptor and epitope functions by chemical modifications at different positions. These combinatorial effects may aid in rapidly avoiding killing by phages and the host immune system, while lowering the typeability of Penner serotyping.

### Concluding remarks.

Here, we demonstrate that MuGENT is applicable for genetic engineering of C. jejuni. In this genetically unstable species, MuGENT specifically provided a feasible and effective approach for editing multiple hypermutable SSRs. Specifically, MuGENT-SSR was performed to uncover the contributions of multiple phase-variable genes to specific phenotypes. Combining MuGENT-SSR with whole-genome SSR analysis (7, 20, 21, 64) may enable comprehensive studies of numerous phase-variable genes in order to decode the phasotypes that determine specific phenotypes and more collective behaviors, such as colonization of animal hosts (19, 29–33).

C. jejuni is currently the only species in which methylation-dependent natural transformation and cotransformation have been demonstrated, although the RAATTY methylation motif and its methyltransferase CtsM are conserved among at least 4 Campylobacter species, including C. jejuni, Campylobacter coli, Campylobacter peloridis, and Campylobacter subantarcticus (39). Campylobacter species other than C. jejuni are known to actually or potentially cause human diseases (65) and generate SSR-mediated phase variation (21) and thus would be candidates for use with MuGENT-SSR.

We also propose that MuGENT-SSR can be utilized to engineer strains suitable for serological typing and vaccines. In C. jejuni NCTC 11168, the majority of polyG/C SSRs are clustered in genomic regions encoding proteins involved in the biosynthesis of cell surface antigenic determinants, including CPS and LOS (8). Thus, phase variation makes these antigens less desirable as serodeterminants and vaccine candidates. Penner serotyping is often utilized to investigate epidemiological associations with GBS (66, 67), but its low typeability is currently problematic (68). The generation of phase-locked strains by MuGENT-SSR may overcome these defects and stabilize their antigenicity, thereby increasing the supply of stable serotyping antisera and vaccines. Furthermore, PPT decoding of Penner serotypes provides a reliable approach for identifying antigenic determinant genes and improving or developing DNA-based typing methods that do not rely on CPS expression (69).

In recent years, several outbreaks of GBS have been observed globally, including the 2019 Peruvian outbreak associated with C. jejuni (70, 71). It is currently established that sialylated LOS molecules play a crucial role in inducing autoimmunity in GBS (13, 15). Therefore, the genes responsible for the production of sialylated LOS, including *wlaN* and *cgtB*, are used as epidemiological markers of C. jejuni strains that can potentially elicit GBS (72, 73). As described above, particular Penner serotypes, including serotype O (antigenic factor HS19), may be also implicated in GBS development (66, 67). However, it remains to be determined experimentally if (or how) these serotypes contribute to the pathogenesis. Thus, functional studies of CPS and LOS using MuGENT-SSR and recently reported animal models (74, 75), together with classical epidemiological typing of these glycans, should help to clarify the overall picture of the mechanisms of C. jejuni*-*induced GBS.

## MATERIALS AND METHODS

### Bacterial culture conditions.

C. jejuni strains used in this study (Table 1) were routinely cultured for 24 to 48 h at 42°C on BHI (Becton, Dickinson) plates containing 1.3% agar (Kyokuto) under microaerophilic conditions using Mitsubishi Anaeropack MicroAero gas generator packs (Mitsubishi Gas). Motility was determined by culturing the cells on BHI plates containing 0.3% agar. For liquid culture, fresh single colonies grown on BHI agar plates were inoculated into 5 ml of BHI broth in 25-ml volumetric test tubes and then cultured overnight at 42°C with reciprocal shaking at 160 rpm in a Taitec Precyto MG-71M-A obligatory anaerobe culture system (Taitec), which can efficiently create a microaerophilic atmosphere by actively aerating gases into each test tube. The aeration conditions in each test tube were set to 5% O_2_, 10% CO_2_, and 85% N_2_ at a constant flow rate (10 ml/min). The antibiotics used were 25 μg/ml chloramphenicol (Cm), 50 μg/ml kanamycin (Km), and 10 μg/ml streptomycin (Sm). 5-Bromo-4-chloro-3-indolyl sulfate (XS) (Sigma) was added to the BHI agar plates at a final concentration of 100 μg/ml.

### DNA manipulation.

PCR amplification of DNA was performed using a LifeECO thermal cycler (version 1.04; Bioer Technology) and Quick Taq HS DyeMix DNA polymerase (Toyobo). Sanger sequencing was entrusted to Fasmac. PCR products were purified using a high pure PCR product purification kit (Roche). Customized oligonucleotide primers (see Table S1 in the supplemental material) were purchased from Fasmac and Hokkaido System Science. Chromosomal and plasmid DNA molecules were extracted with a DNeasy blood and tissue kit (Qiagen) and a high pure plasmid isolation kit (Roche), respectively.

10.1128/mBio.01401-21.5 TABLE S1 Primers used in this study. Download Table S1, PDF file, 0.10 MB. Copyright © 2021 Yamamoto et al. 2021 Yamamoto et al. https://creativecommons.org/licenses/by/4.0/ This content is distributed under the terms of the Creative Commons Attribution 4.0 International license.

### Construction of pSYC-*cat* and pSYC-*kan*.

The pSYC-*cat* and pSYC-*kan* plasmids (Table 1) were constructed by Fasmac as follows: DNA fragments containing the Cm resistance gene (*cat*) (GenBank accession number M35190.1; nucleotides 1 to 1,034) and the Km resistance gene (*kan*) (GenBank accession number M26832.1; nucleotides 1 to 1,426) from C. coli (76, 77) were synthesized and cloned into the EcoRV site of pUCFa (Fasmac) to generate pSYC-*cat* and pSYC-*kan*, respectively.

### Natural transformation of C. jejuni cells using PCR products.

Genetically engineered C. jejuni strains were constructed by performing natural transformation (39) optimized to use PCR products as donor DNA. Briefly, the procedures include the following three processes: (i) PCR amplification of donor DNA with methylation sites, (ii) methylation of the amplified DNA, and (iii) transformation of C. jejuni with methylated DNA.

### PCR amplification of donor DNA with methylation sites.

The donor DNA fragments used for natural transformation were amplified using specific primers and templates. The typical amplified fragment contained an antibiotic resistance gene (or mutation) with 5′- and 3′-flanking regions homologous to the target site and an EcoRI-recognition sequence (GAATTC) on both sides. DNA was amplified via splicing by overlap extension PCR, which includes a two-step amplification process (78, 79). The first step independently generated two or three fragments with overlapping sequences. In the second step, the splicing by overlap extension PCR fragments amplified in the first step are ligated. The thermal cycling program was as follows: 94°C for 5 min, followed by 30 cycles of 94°C for 30 s, 55°C for 30 s, and 68°C for 1 min per kb. Table S2 in the supplemental material shows the specific combinations of template DNA and primer pairs used for PCR amplification.

### Methylation of donor DNA.

DNA methylation was performed using a 25-μl mixture containing 0.5 or 1.5 pmol DNA, EcoRI methyltransferase reaction buffer (New England BioLabs; 50 mM Tris-HCl, 50 mM NaCl, and 10 mM ethylenediaminetetraacetic acid [pH 8.0]), 80 μM *S*-adenosylmethionine (New England Biolabs), and 40 units of EcoRI methyltransferase (New England BioLabs). After incubation for 2.5 h at 37°C, the reaction was terminated by incubation for 20 min at 65°C. Methylated DNA was purified using the high pure PCR product purification kit and dissolved in 100 μl H_2_O.

### Natural transformation.

Overnight cultures of C. jejuni cells were diluted 1/50 in 5 ml BHI broth and grown until they reached an optical density and 600 nm (OD_600_) of approximately 0.15, which was measured by the MP1200 spectrometer (Seikosya), or grown for 5 to 6 h. Each culture (900 μl) was mixed with methylated DNA (100 μl) and then incubated statically overnight in a 25-ml volumetric plastic test tube. On the following day, 10-fold serial dilutions of the culture were spread onto BHI agar plates, with or without antibiotics. The transformation frequency was defined as the number of antibiotic-resistant CFU divided by the total number of CFU.

### Evaluation of natural cotransformation in C. jejuni.

Natural cotransformation in C. jejuni was examined using two different PCR fragments, namely, Δ*flaA*::*kan* and *rpsL*^+^ (see Fig. 2 and Table S2). The former fragment (Δ*flaA*::*kan*-A and Δ*flaA*::*kan*-B, used for the recipients SY1003 and SY2003, respectively) had 1,000-bp regions of homology, whereas the latter had 1,000-bp or 2,000-bp regions of homology (*rpsL*^+^-8-A or *rpsL*^+^-9-A and *rpsL*^+^-8-B or *rpsL*^+^-9-B, used for SY1003 and SY2003, respectively). The Δ*flaA*::*kan* and *rpsL*^+^ fragments were independently methylated and then mixed for purification as described above. After transformation of SY1003 and SY2003 with these methylated DNA fragments, the reactions were spread onto BHI agar plates containing Km. One hundred Km^r^ transformants were plated onto BHI agar plates containing Sm. The cotransformation frequency (%) was calculated as follows: 100 × number of Sm^s^ CFU in 100 Km^r^ CFU.

### MuGENT-SSR.

MuGENT-SSR was performed with C. jejuni cells, as follows. After independently methylating multiple unselected fragments (each 1.5 pmol), including eight fragments used for editing of the eight polyG sites in the biosynthetic LOS and CPS gene clusters in NCTC11168, as well as a single selected fragment (either Δ*flaA*::*kan*-A or Δ*flaA*::*cat*-A, each 0.5 pmol), a mixture of these fragments was prepared as described above. Unselected fragments had 1,500- to 2,000-bp regions of homology, whereas the selected fragment had a 1,000-bp region of homology (Tables S2 and S3 in the supplemental material). After transformation with the DNA mixture, the bacteria were plated onto BHI agar plates containing the antibiotic corresponding to the resistance protein encoded in the selected fragment. Colonies on selective agar plates were used for MASC PCR (described below). The remaining colonies (3,000 to 5,000 CFU) were suspended in BHI broth and diluted in 5 ml BHI broth to an OD_600_ of 0.05. After the cells were grown to an OD_600_ of 0.15, the next cycle of MuGENT was performed using unselected fragments and a selected fragment with a different resistance gene from that used in the previous cycle. MuGENT cycles were repeated until editing was completed. Genome editing was ultimately verified by nucleotide sequencing using specific primers (see the PPT-Seq section below). If necessary, the Δ*flaA* mutation maintained during MuGENT was reverted to the wild-type allele by transformation using the *flaA*^+^ fragment with a 1,000-bp region of homology (see Fig. 2 and Table S2, *flaA*^+^-A). An aliquot of the transformation (50 to 100 μl) was spotted onto a BHI plate containing 0.3% agar to select motile variants. Single colonies of the variants were tested for sensitivity to the antibiotic used for selection.

10.1128/mBio.01401-21.7 TABLE S3 Specific combinations of donor DNA molecules and recipient strains used for natural transformation. Download Table S3, PDF file, 0.08 MB. Copyright © 2021 Yamamoto et al. 2021 Yamamoto et al. https://creativecommons.org/licenses/by/4.0/ This content is distributed under the terms of the Creative Commons Attribution 4.0 International license.

### MASC PCR.

During each cycle of MuGENT, 40 colonies were inoculated in 100 μl of BHI broth in a 96-well plate and incubated overnight. Each PCR mixture (20 μl) contained 2 μl of the bacterial culture, 2 μl of 2.5 μM primer mix (one of four primer mixes, including Mix ON1, Mix ON2, Mix OFF1, and Mix OFF2; see Table S4 in the supplemental material), 6 μl of H_2_O, and 10 μl of Quick Taq high sensitivity (HS) DyeMix. The following thermal cycling program was used for MASC PCR: 94°C for 5 min, followed by 35 cycles of 94°C for 30 s, 62.6°C for 30 s, and 68°C for 3 min. Several single colonies were isolated from positive colonies (to eliminate contamination by unedited clones) and further assessed for genome editing by MASC PCR.

10.1128/mBio.01401-21.8 TABLE S4 Primer mixes used for MASC PCR. Download Table S4, PDF file, 0.07 MB. Copyright © 2021 Yamamoto et al. 2021 Yamamoto et al. https://creativecommons.org/licenses/by/4.0/ This content is distributed under the terms of the Creative Commons Attribution 4.0 International license.

### PPT-Seq.

The eight phase-variable genes located in the LOS- and CPS-biosynthetic gene clusters in NCTC11168 were amplified by PCR, and the repeat numbers of the polyG SSR tracts were determined by nucleotide sequencing. Table S5 in the supplemental material shows the specific combinations of target genes and primers used for PPT-Seq.

10.1128/mBio.01401-21.9 TABLE S5 Specific combinations of target genes and primers in PPT-Seq. Download Table S5, PDF file, 0.05 MB. Copyright © 2021 Yamamoto et al. 2021 Yamamoto et al. https://creativecommons.org/licenses/by/4.0/ This content is distributed under the terms of the Creative Commons Attribution 4.0 International license.

### Penner serotyping.

A single colony was streaked on the surface of a horse blood agar plate (Kyokuto) and incubated for 48 h at 42°C to determine the Penner serotype (80). After the resulting colonies grown on the plate were suspended in 250 μl of 0.9% NaCl, serotyping was performed using Campylobacter antisera (Denka), including a commercial 25 antisera set and a reagent for preparing sensitized blood cells (Denka) according to the manufacturer’s instructions. Phenotypic stability was assessed by repeating the subculture cycle and serotyping. Serotyping was performed using six single colonies for each cycle.

### Measurement of mutation rates.

The mutation rates of the phase-variable *cj1426* gene and its phase-locked variants were determined as described previously (81), with some modifications. C. jejuni strains carrying *astA* to *cj1426* translational fusions were streaked onto BHI agar plates containing XS. During growth on the medium, blue colonies were in the *cj1426*-ON phase, whereas white colonies were in the *cj1426*-OFF phase. Single blue or white colonies picked using a micropipette tip were resuspended in 500 μl BHI, after which 250 μl of 10^4^-, 10^5^-, and 10^6^-fold dilutions were spread onto BHI agar plates containing XS. The number of variant colonies that switched to different phases and the total numbers of colonies were counted. Ten independent single colonies were examined for each strain and phase. To estimate the mutation rate, the total number of colonies was averaged for all single colonies tested, and the median value for the frequency of variants per colony was calculated. The mutation rate was calculated using the following equation: μ=0.4343f/log(Nμ), where μ is the mutation rate, f is the median frequency, and N is the average population size (82). The μ value was determined by solving the equation using the Goal Seek function in Microsoft Excel.

10.1128/mBio.01401-21.10 TABLE S6 Primer sets used for allele-specific PCR and sequencing of *cj1426*::*astA* translational fusions. Download Table S6, PDF file, 0.07 MB. Copyright © 2021 Yamamoto et al. 2021 Yamamoto et al. https://creativecommons.org/licenses/by/4.0/ This content is distributed under the terms of the Creative Commons Attribution 4.0 International license.
