# Supplementary material for: Stabilizing Genetically Unstable Simple Sequence Repeats in the Campylobacter jejuni Genome by Multiplex Genome Editing: a Reliable Approach for Delineating Multiple Phase-Variable Genes
Source: mBio. 2021 Aug 24;12(4):e01401-21. doi: 10.1128/mBio.01401-21 (PMC8437040; doi:10.1128/mBio.01401-21)
Supplement: TABLE S2 [file mbio.01401-21-st002.pdf]

**Table S2. Specific combinations of template DNA and primers used to amplify donor DNA templates**

| First-step PCR | | Second-step PCR | | Generated PCR product[a] (length of homologous region) |
|---|---|---|---|---|
| Template | Primers | Template | Primers | |
| Chromosomal DNA NCTC11168 | cj1339c(flaA)-f1E and cj1339c-cat-r1 | First-step PCR products | cj1339c(flaA)-f1E and cj1339c(flaA)-r2E | Δ*flaA*::*cat*-A (1 kb) |
| pSYC-*cat* | c-cat-f1 and c-cat-r2 | | | |
| Chromosomal DNA NCTC11168 | cat-cj1339c(flaA)-f2 and cj1339c(flaA)-r2E | | | |
| Chromosomal DNA NCTC11168 | cj1339c(flaA)-f1E and cj1339c-kan-r1 | First-step PCR products | cj1339c(flaA)-f1E and cj1339c(flaA)-r1E | Δ*flaA*::*kan*-A (1 kb) |
| pSYC-*kan* | c-kan-f1 and c-kan-r1 | | | |
| Chromosomal DNA NCTC11168 | kan-cj1339c(flaA)-f1 and cj1339c(flaA)-r1E | | | |
| Chromosomal DNA NCTC11168 | cj1339c(flaA)-f1E and cj1339c(flaA)-r2E | / | / | *flaA*$^+$-A (1 kb) |
| Chromosomal DNA NCTC11168 | cj1673c-f1000E and 176_1669-pUCFa-r1 | First-step PCR products | cj1673c-f1000E and cj1673c-r1000E | Δ*recA*::*cat*-A (1 kb) |
| pSYC-*cat* | pUCFa-f1 and pUCFa-r1 | | | |

| | | | | |
|---|---|---|---|---|
| Chromosomal DNA NCTC11168 | pUCFa-176_1669-f1 and cj1673c-r1000E | | | |
| Chromosomal DNA SYC1003 | 176rpsLmt-f50E and 176rpsLmt-r50E | | | *rpsL*$^{K88R}$-1 (50 b) |
| Chromosomal DNA SYC1003 | 176rpsLmt-f100E and 176rpsLmt-r100E | | | *rpsL*$^{K88R}$-2 (100 b) |
| Chromosomal DNA SYC1003 | 68rpsLmt-f500 and 176rpsLmt-r500 | | | *rpsL*$^{K88R}$-3-A (500 b) |
| Chromosomal DNA SYC1003 | 68rpsLmt-f500E and 176rpsLmt-r500 | | | *rpsL*$^{K88R}$-4-A (500 b) |
| Chromosomal DNA SYC1003 | 68rpsLmt-f500E and 176rpsLmt-r500E | | | *rpsL*$^{K88R}$-5-A (500 b) |
| Chromosomal DNA SYC1003 | 68rpsLmt-f1000 and 176rpsLmt-r1000 | | | *rpsL*$^{K88R}$-6-A (1 kb) |
| Chromosomal DNA SYC1003 | 68rpsLmt-f1000E and 176rpsLmt-r1000 | | | *rpsL*$^{K88R}$-7-A (1 kb) |
| Chromosomal DNA NCTC11168 | 68rpsLmt-f1000E and rpsL(CJ0491)-StmR-R2 | First-step PCR products | 68rpsLmt-f1000E and 176rpsLmt-r1000E | *rpsL*$^{K88R}$-8-A (1 kb) |
| Chromosomal DNA NCTC11168 | rpsL(CJ0491)-StmR-F2 and 176rpsLmt-r1000E | | | |
| Chromosomal DNA SYC1003 | 68rpsLmt-f2000E and 176rpsLmt-r2000E | | | *rpsL*$^{K88R}$-9-A (2 kb) |
| Chromosomal DNA | 68rpsLmt-f1000E and | | | *rpsL*$^{+}$-8-A (1 kb) |

| | | | | |
|---|---|---|---|---|
| NCTC11168 | 176rpsLmt-r1000E | | | |
| Chromosomal DNA NCTC11168 | 68rpsLmt-f2000E and 176rpsLmt-r2000E | | | *rpsL*$^+$-9-A (2 kb) |
| Chromosomal DNA NCTC11168 | cj1426c-f1E and cj1426c-ON-r1 | First-step PCR products | cj1426c-f1E and cj1426c-r1E | *cj1426*$^{ON}$ (2 kb) |
| Chromosomal DNA NCTC11168 | cj1426c-ON-f1 and cj1426c-r1E | | | |
| Chromosomal DNA NCTC11168 | cj1426c-f1E and cj1426c-OFF(-1)-r2 | First-step PCR products | cj1426c-f1E and cj1426c-r1E | *cj1426*$^{OFF}$ (2 kb) |
| Chromosomal DNA NCTC11168 | cj1426c-OFF(-1)-f2 and cj1426c-r1E | | | |
| Chromosomal DNA NCTC11168 | cj1429c-f3E and cj1429c-ON-r2 | First-step PCR products | cj1429c-f3E and cj1429c-r1E | *cj1429*$^{ON}$ (2 kb) |
| Chromosomal DNA NCTC11168 | cj1429c-ON-f1 and cj1429c-r1E | | | |
| Chromosomal DNA NCTC11168 | cj1429c-f3E and cj1429c-OFF(-1)-r2 | First-step PCR products | cj1429c-f3E and cj1429c-r1E | *cj1429*$^{OFF}$ (2 kb) |
| Chromosomal DNA NCTC11168 | cj1429c-OFF(-1)-f2 and cj1429c-r1E | | | |
| Chromosomal DNA NCTC11168 | cj1139c-f2E and cj1139c-ON-r1 | First-step PCR products | cj1139c-f2E and cj1139c-r2E | *cj1139*$^{ON}$ (2 kb) |
| Chromosomal DNA NCTC11168 | cj1139c-ON-f1 and cj1139c-r2E | | | |

| | | | | |
|---|---|---|---|---|
| Chromosomal DNA NCTC11168 | cj1139c-f2E and cj1139c-OFF(-1)-r2 | First-step PCR products | cj1139c-f2E and cj1139c-r2E | *cj1139*$^{\mathrm{OFF}}$ (2 kb) |
| Chromosomal DNA NCTC11168 | cj1139c-OFF(-1)-f2 and cj1139c-r2E | | | |
| Chromosomal DNA NCTC11168 | cj1420c-f1E and cj1420c -ON-r1 | First-step PCR products | cj1420c-f1E and cj1420c-r1E | *cj1420*$^{\mathrm{ON}}$ (2 kb) |
| Chromosomal DNA NCTC11168 | cj1420c -ON-f1 and cj1420c-r1E | | | |
| Chromosomal DNA NCTC11168 | cj1420c-f1E and cj1420c -OFF(-1)-r2 | First-step PCR products | cj1420c-f1E and cj1420c-r1E | *cj1420*$^{\mathrm{OFF}}$ (2 kb) |
| Chromosomal DNA NCTC11168 | cj1420c -OFF(-1)-f2 and cj1420c-r1E | | | |
| Chromosomal DNA NCTC11168 | cj1145c-f1E and cj1145c -ON-r1 | First-step PCR products | cj1145c-f1E and cj1145c-r1E | *cj1144*$^{\mathrm{ON}}$ (2 kb) |
| Chromosomal DNA NCTC11168 | cj1145c -ON-f1 and cj1145c -r1E | | | |
| Chromosomal DNA NCTC11168 | cj1145c-f1E and cj1145c -OFF(-1)-r2 | First-step PCR products | cj1145c-f1E and cj1145c-r1E | *cj1144*$^{\mathrm{OFF}}$ (2 kb) |
| Chromosomal DNA NCTC11168 | cj1145c - OFF(-1)-f2 and cj1145c -r1E | | | |
| Chromosomal DNA NCTC11168 | cj1437c-f1E and cj1437c -ON-r1 | First-step PCR products | cj1437c-f1E and cj1437c-r1E | *cj1437*$^{\mathrm{ON}}$ (2 kb) |
| Chromosomal DNA | cj1437c -ON-f1 and | | | |

| | | | | |
|---|---|---|---|---|
| NCTC11168 | cj1437c -r1E | | | |
| Chromosomal DNA NCTC11168 | cj1437c-f1E and cj1437c -OFF(-1)-r3 | First-step PCR products | cj1437c-f1E and cj1437c-r1E | *cj1437*$^{OFF}$ (2 kb) |
| Chromosomal DNA NCTC11168 | cj1437c -OFF(-1)-f3 and cj1437c -r1E | | | |
| Chromosomal DNA NCTC11168 | cj1422c-f1E and cj1421/22cc -ON-r1 | First-step PCR products | cj1422c-f1E and cj1422c-r2E | *cj1422*$^{ON}$ (2 kb) |
| Chromosomal DNA NCTC11168 | cj1421/22cc -ON-f1 and cj1422c-r2E | | | |
| Chromosomal DNA NCTC11168 | cj1422c-f1E and cj1421/22cc -OFF(-1)-r2 | First-step PCR products | cj1422c-f1E and cj1422c-r2E | *cj1422*$^{OFF}$ (2 kb) |
| Chromosomal DNA NCTC11168 | cj1421/22cc -OFF(-1)-f2 and cj1422c-r2E | | | |
| Chromosomal DNA NCTC11168 | cj1422c-f2E and cj1421/22cc -ON-r1 | First-step PCR products | cj1422c-f2E and cj1422c-r1E | *cj1421*$^{ON}$ (2 kb) |
| Chromosomal DNA NCTC11168 | cj1421/22cc -ON-f1 and cj1422c-r1E | | | |
| Chromosomal DNA NCTC11168 | cj1422c-f2E and cj1421/22cc -OFF(-1)-r2 | First-step PCR products | cj1422c-f2E and cj1422c-r1E | *cj1421*$^{OFF}$ (2 kb) |
| Chromosomal DNA NCTC11168 | cj1421/22cc -OFF(-1)-f2 and cj1422c-r1E | | | |

| | | | | |
|---|---|---|---|---|
| Chromosomal DNA 81-176 | cjj81176_1339-f1E and flaA81176-cat-r1 | First-step PCR products | cjj81176_1339-f1E and cjj81176_1339-r1E | Δ*flaA*::*cat*-B (1 kb) |
| pSYC-*cat* | c-cat-f1 and c-cat-r2 | | | |
| Chromosomal DNA 81-176 | cat-flaA81176-f2 and cjj81176_1339-r1E | | | |
| Chromosomal DNA 81-176 | cjj81176_1339-f1E and flaA81176-kan-f1 | First-step PCR products | cjj81176_1339-f1E and cjj81176_1339-r1E | Δ*flaA*::*kan*-B (1 kb) |
| pSYC-*kan* | c-kan-f1 and c-kan-r1 | | | |
| Chromosomal DNA 81-176 | kan-flaA81176-f1 and cjj81176_1339-r1E | | | |
| Chromosomal DNA 81-176 | cjj81176_1339-f1E and cjj81176_1339-r1E | | | *flaA*$^{+}$-B (1 kb) |
| Chromosomal DNA 81-176 | 176_1669-f1000E and 176_1669-pUCFa-r1 | First-step PCR products | 176_1669-f1000E and 176_1669-r1000E | Δ*recA*::*cat*-B (1 kb) |
| pSYC-*cat* | pUCFa-f1 and pUCFa-r1 | | | |
| Chromosomal DNA 81-176 | pUCFa-176_1669-f1 and 176_1669-r1000E | | | |
| Chromosomal DNA SYC2003 | 176rpsLmt-f500 and 176rpsLmt-r500 | | | *rpsL*$^{K88R}$-3-B (500 b) |
| Chromosomal DNA SYC2003 | 176rpsLmt-f500E and 176rpsLmt-r500 | | | *rpsL*$^{K88R}$-4-B (500 b) |
| Chromosomal DNA | 176rpsLmt-f500E and | | | *rpsL*$^{K88R}$-5-B (500 b) |

| | | | | |
|---|---|---|---|---|
| SYC2003 | 176rpsLmt-r500E | | | |
| Chromosomal DNA SYC2003 | 176rpsLmt-f1000 and 176rpsLmt-r1000 | | | *rpsL*$^{K88R}$-6-B (1 kb) |
| Chromosomal DNA SYC2003 | 176rpsLmt-f1000E and 176rpsLmt-r1000 | | | *rpsL*$^{K88R}$-7-B (1 kb) |
| Chromosomal DNA 81-176 | 176rpsLmt-f1000E and rpsL(CJ0491)-StmR-R2 | First-step PCR products | 176rpsLmt-f1000E and 176rpsLmt-r1000E | *rpsL*$^{K88R}$-8-B (1 kb) |
| Chromosomal DNA 81-176 | rpsL(CJ0491)-StmR-F2 and 176rpsLmt-r1000E | | | |
| Chromosomal DNA SYC2003 | 176rpsLmt-f2000E and 176rpsLmt-r2000E | | | *rpsL*$^{K88R}$-9-B (1 kb) |
| Chromosomal DNA 81-176 | 176rpsLmt-f1000E and 176rpsLmt-r1000E | | | *rpsL*$^{+}$-8-B (1 kb) |
| Chromosomal DNA 81-176 | 176rpsLmt-f2000E and 176rpsLmt-r2000E | | | *rpsL*$^{+}$-9-B (2 kb) |
| Chromosomal DNA SYC1P255 | cj1426c-f1E and cj1426c-astA-r2 | First-step PCR products | cj1426c-f1E and cj1426c-r1E | *cj1426*$^{ON}$::*astA* (2 kb) |
| Chromosomal DNA 81-176 | astA-f2 and astA-r2 | | | |
| Chromosomal DNA NCTC11168 | astA-cj1426c-f2 and cj1426c-r1E | | | |
| Chromosomal DNA SYC1007 | cj1426c-f1E and cj1426c-WT-r1 | First-step PCR products | cj1426c-f1E and cj1426c-r1E | *cj1426*::*astA* (2 kb) |

| | | | | |
|---|---|---|---|---|
| Chromosomal DNA SYC1007 | cj1426c-WT-f1 and cj1426c-r1E | | | |
| Chromosomal DNA SYC1007 | cj1426c-f1E and cj1426c-OFF(-1)-r2 | First-step PCR products | cj1426c-f1E and cj1426c-r1E | *cj1426*$^{\mathrm{OFF}}$::*astA* (2 kb) |
| Chromosomal DNA SYC1007 | cj1426c-OFF(-1)-f2 and cj1426c-r1E | | | |

[a]PCR products labelled "A" (including Δ*flaA*::*cat*-A, Δ*flaA*::*kan*-A, *flaA*$^{+}$-A, Δ*recA*::*cat*-A, *rpsL*$^{\mathrm{K88R}}$-3-A, *rpsL*$^{\mathrm{K88R}}$-4-A, *rpsL*$^{\mathrm{K88R}}$-5-A, *rpsL*$^{\mathrm{K88R}}$-6-A, *rpsL*$^{\mathrm{K88R}}$-7-A, *rpsL*$^{\mathrm{K88R}}$-8-A, *rpsL*$^{\mathrm{K88R}}$-9-A, *rpsL*$^{+}$-8-A and *rpsL*$^{+}$-9-A) were specifically used for NCTC11168, while those labelled "B" (including Δ*flaA*::*cat*-B, Δ*flaA*::*kan*-B, *flaA*$^{+}$-B, Δ*recA*::*cat*-B, *rpsL*$^{\mathrm{K88R}}$-3-B, *rpsL*$^{\mathrm{K88R}}$-4-B, *rpsL*$^{\mathrm{K88R}}$-5-B, *rpsL*$^{\mathrm{K88R}}$-6-B, *rpsL*$^{\mathrm{K88R}}$-7-B, *rpsL*$^{\mathrm{K88R}}$-8-B, *rpsL*$^{\mathrm{K88R}}$-9-B, *rpsL*$^{+}$-8-B and *rpsL*$^{+}$-9-B) were specifically for 81-176.
