# Supplementary material for: Stabilizing Genetically Unstable Simple Sequence Repeats in the Campylobacter jejuni Genome by Multiplex Genome Editing: a Reliable Approach for Delineating Multiple Phase-Variable Genes
Source: mBio. 2021 Aug 24;12(4):e01401-21. doi: 10.1128/mBio.01401-21 (PMC8437040; doi:10.1128/mBio.01401-21)
Supplement: FIG S2 [file mbio.01401-21-sf002.pdf]

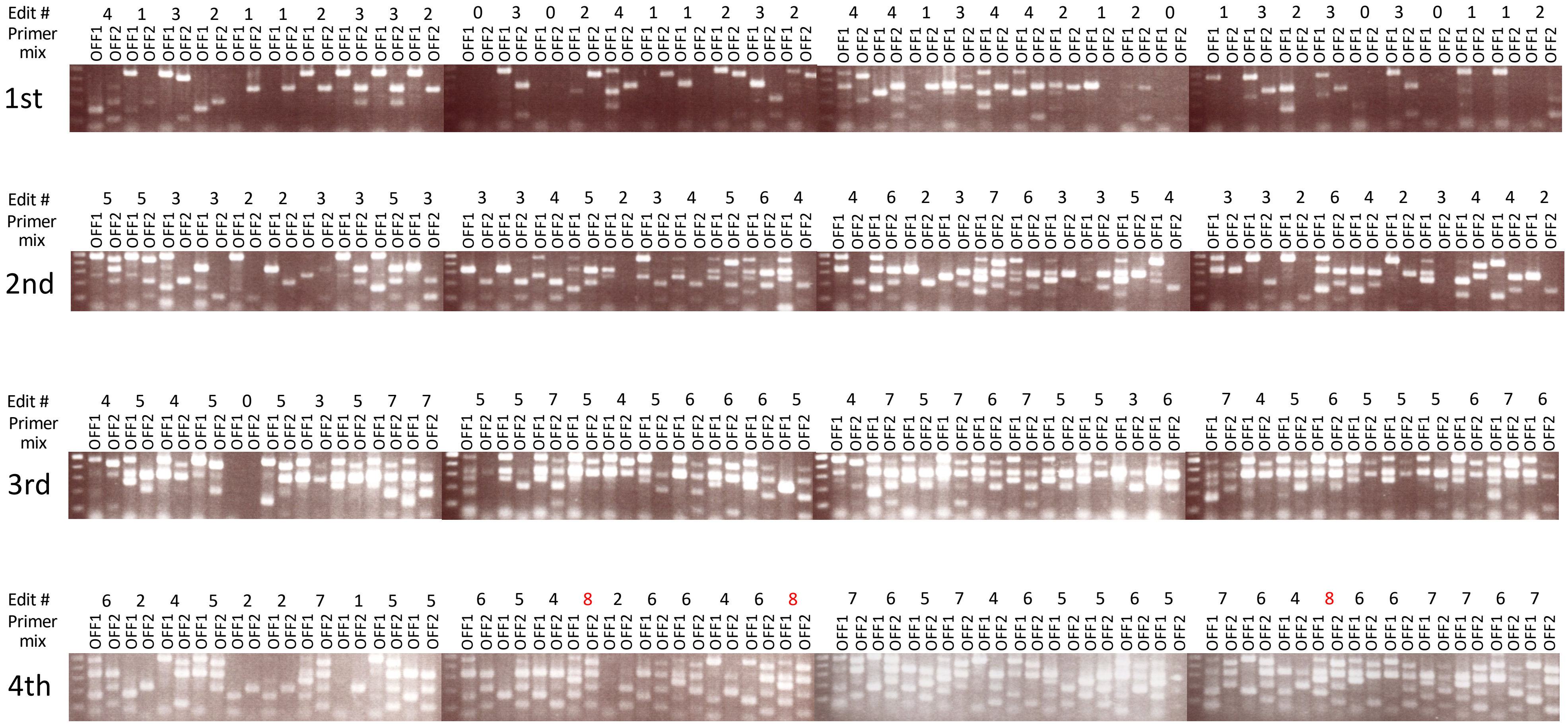

Fig. S2. MASC PCR of transformants following successive cycles of MuGENT-SSR. Forty transformants per cycle were subjected to MASC PCR using the primer mixes, Mix OFF1 and Mix OFF2 (Table S4). Edit # indicates the number of genome edits of each transformant. Transformants with all eight genome edits are shown in red.
