# Supplementary material for: Stabilizing Genetically Unstable Simple Sequence Repeats in the Campylobacter jejuni Genome by Multiplex Genome Editing: a Reliable Approach for Delineating Multiple Phase-Variable Genes
Source: mBio. 2021 Aug 24;12(4):e01401-21. doi: 10.1128/mBio.01401-21 (PMC8437040; doi:10.1128/mBio.01401-21)
Supplement: FIG S3 [file mbio.01401-21-sf003.pdf]

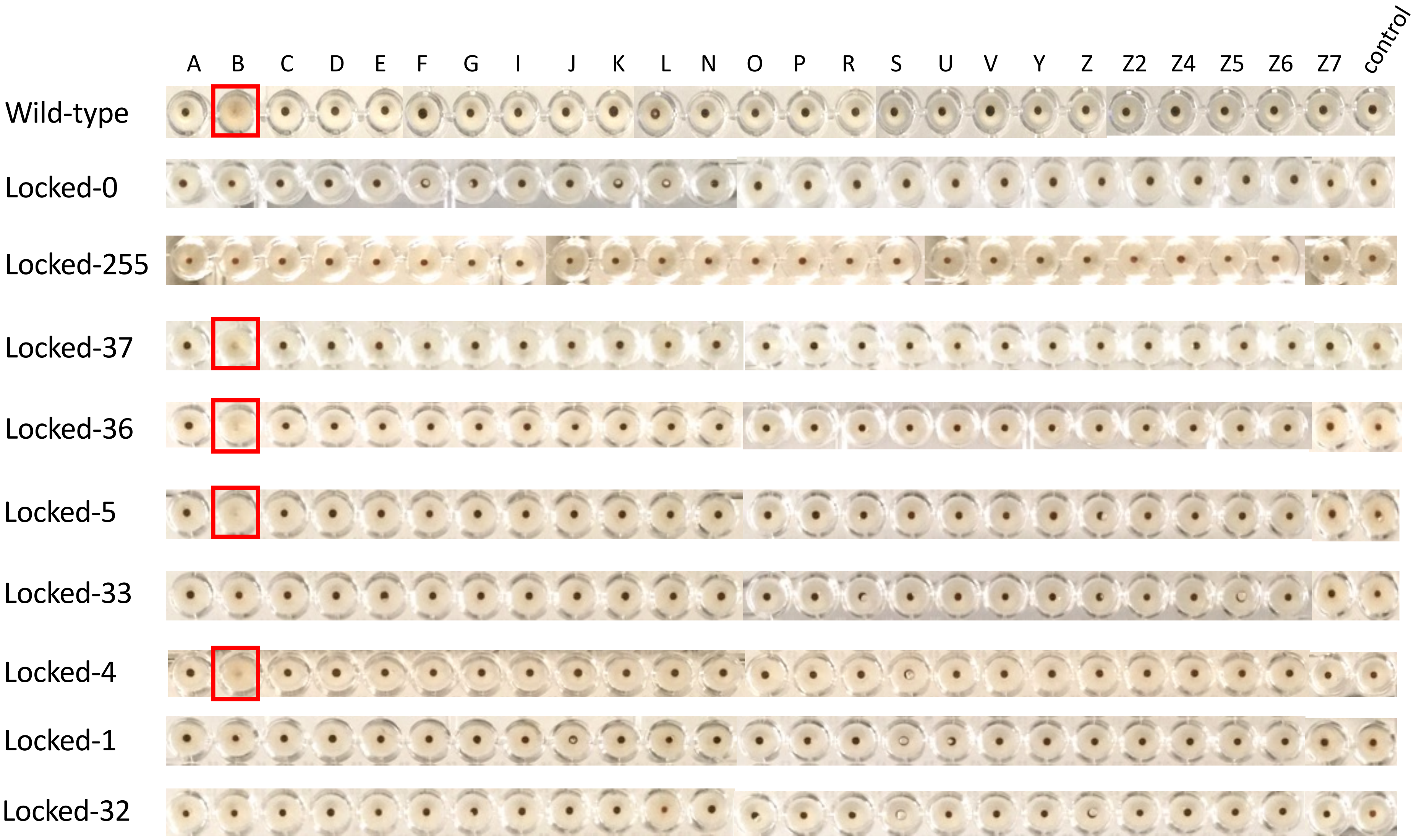

Fig. S3. Penner serotyping of phase-locked NCTC11168 variants. See Fig. 5 for strains used. Wells typed as “B” are marked with red squares.
