# Supplementary material for: Stabilizing Genetically Unstable Simple Sequence Repeats in the Campylobacter jejuni Genome by Multiplex Genome Editing: a Reliable Approach for Delineating Multiple Phase-Variable Genes
Source: mBio. 2021 Aug 24;12(4):e01401-21. doi: 10.1128/mBio.01401-21 (PMC8437040; doi:10.1128/mBio.01401-21)
Supplement: FIG S4 [file mbio.01401-21-sf004.pdf]

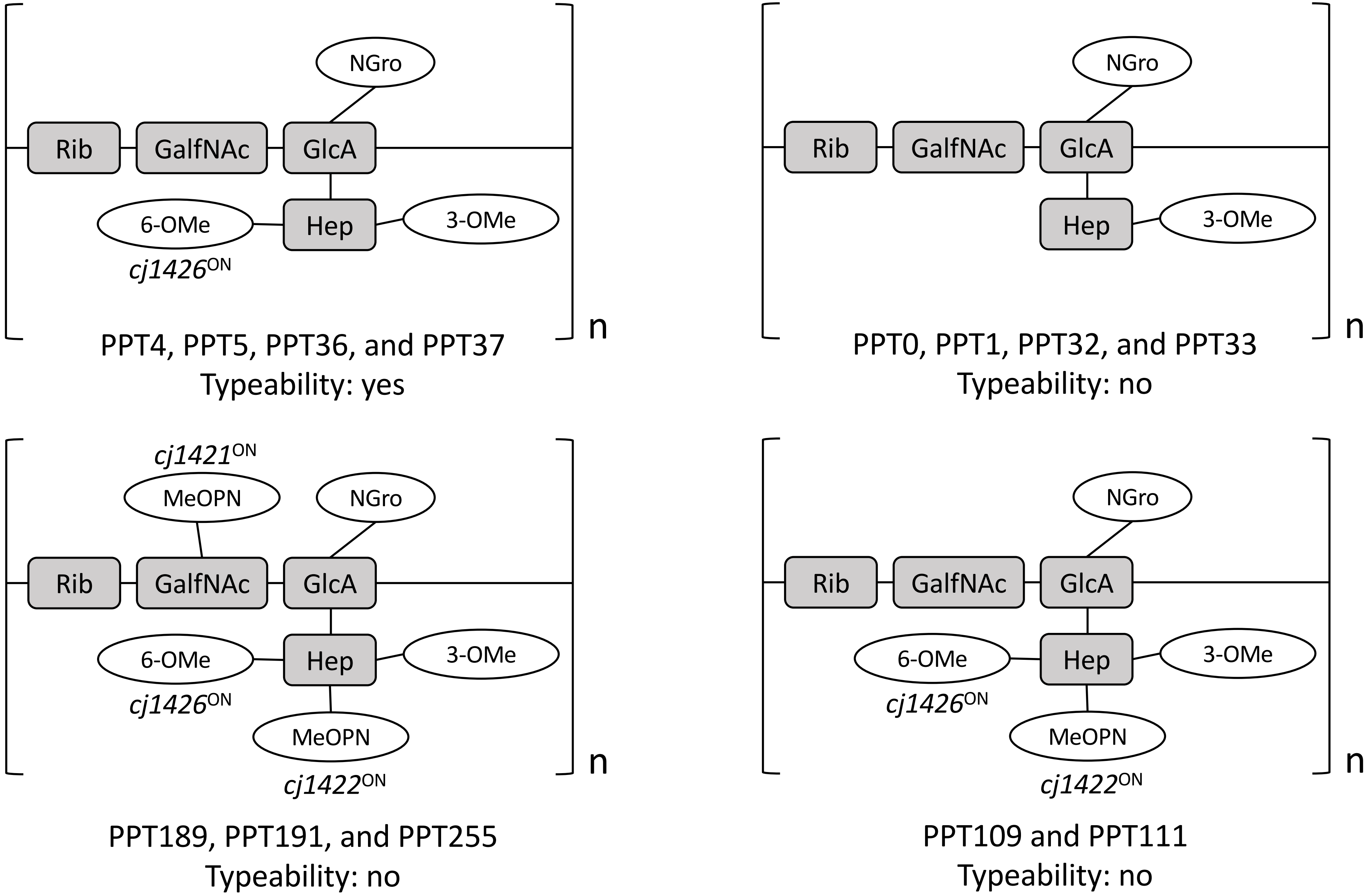

Fig. S4. Putative modification patterns of the CPS repeat unit in naturally occurring and phase-locked NCTC11168 variants. Figures were modified from illustrations published in Sternberg *et al*, *J Mol Biol* 2013 (425) 186-197. Abbreviations for the sugars and modifications are as follows: MeOPN, O-methyl-phosphoramidate; NGro, N-glycerol; Rib, ribose; GalfNAc, N-acetylgalactosamine in the furanose configuration; GlcA, glucuronic acid; 6-OMe, 6-O-Methyl; Hep, heptose, 3-OMe, 3-O-methyl.
