# Supplementary material for: Stabilizing Genetically Unstable Simple Sequence Repeats in the Campylobacter jejuni Genome by Multiplex Genome Editing: a Reliable Approach for Delineating Multiple Phase-Variable Genes
Source: mBio. 2021 Aug 24;12(4):e01401-21. doi: 10.1128/mBio.01401-21 (PMC8437040; doi:10.1128/mBio.01401-21)
Supplement: TABLE S1 [file mbio.01401-21-st001.pdf]

Table S1. Primers used in this study

Name	Sequence[a] (5′–3′)
Used for natural transformation	
176_1669-f1000E	GGGGAATTCCAACTAAGGTACTTTCGTCC
176_1669-pUCFa-r1	GGAAGAGCGTCGTTAAGGGAAAGGCAGCGTCTAGAGATTTTC
176_1669-r1000E	GGGGAATTCGGTTTAATAAGCACAGCATTTGC
176rpsLmt-f1000	GCGTTCTAAAGAAAGAATTATTATCCAAGC
176rpsLmt-f1000E	GGGGAATTCGCGTTCTAAAGAAAGAATTATTATCCAAGC
176rpsLmt-f100E	GGGGAATTCGTAAGACTTACTAGTGGCTTTG
176rpsLmt-f2000E	GGGGAATTCCGGCGATTTTACTTGTGGAG
176rpsLmt-f500	TAGCGGGTAAATTTGACTACTTAGAAG
176rpsLmt-f500E	GGGGAATTCTAGCGGGTAAATTTGACTACTTAGAAG
176rpsLmt-f50E	GGGGAATTCTCATAACTTGCAAGAACACAGC
176rpsLmt-r1000	TGAACAAAATACCGCAACAGCACCATC
176rpsLmt-r1000E	GGGGAATTCTGAACAAAATACCGCAACAGCACCATC
176rpsLmt-r100E	GGGGAATTCGCGTTTAGCACCATATTTAGAACG
176rpsLmt-r2000E	GGGGAATTCCATCTGTAGAAACTCTAAAACTTGG
176rpsLmt-r500	AACGAATCGCCAAAGCTTGTTGTCTAGC
176rpsLmt-r500E	GGGGAATTCAACGAATCGCCAAAGCTTGTTGTCTAGC
176rpsLmt-r50E	GGGGAATTCCTGTATCAAGAGCACCACGAAC
68rpsLmt-f1000	GCGATTGTAGAATGTGGTGGAAAAATCAC
68rpsLmt-f1000E	GGGGAATTCGCGATTGTAGAATGTGGTGGAAAAATCAC
68rpsLmt-f2000E	GGGGAATTCGAAAGTTTATCCCACATAATGGAG

68rpsLmt-f500	AGTAATTTCTTAAAATCAATTTTGG
68rpsLmt-f500E	GGGGAATTCAGTAATTTCTTAAAATCAATTTTGG
astA-cj1426c-f2	GCAATGCCTTTTAGTGTTGATCAAGCATTCAATCCTAAAAAATAATCAGAGAAAA AGATAGGGGAG
astA-f2	AGACTTAGCAAAACTCTTTGTATGGCACTTTTGGCGGGC
astA-r2	TTATTTTTTAGGATTGAATGCTTGATCAACACTAAAAGGCATTGC
c-cat-f1	ATTCCCACAACGCCGGAAAC
c-cat-r2	AATGAAGCTCCGCAGGACGC
c-kan-f1	GATAAACCCAGCGAACCATT
c-kan-r1	GCTTTTTAGACATCTAAATCTAGG
cat-cj1339c(flaA)-f2	GCGTCCTGCGGAGCTTCATTGCTGCAATATATACAAATCC
cat-flaA81176-f2	GCGTCCTGCGGAGCTTCATTGCAATGGCTCAAGCAAATTC
cj1139c-OFF(-1)-f2	GGATATGGGTGGAGGCGTAAAATTGATTTGTTG
cj1139c-OFF(-1)-r2	CAACAAATCAATTTTACGCCTCCACCCATATCC
cj1139c-ON-f1	GGATATGGGTGGCGGAGGTAAAATTGATTTGTTG
cj1139c-ON-r1	CAACAAATCAATTTTACCTCCGCCACCCATATCC
cj1139c-f2E	GGGGAATTCTAATCTTACTAAGTGCGATGG
cj1139c-r2E	GGGGAATTCAGGTGAAGCTTGATTTTTACC
cj1145c-OFF(-1)-f2	CTTTATCTTAAAAAAAAAGGAGGCTATGGGTAGATCTTG
cj1145c-OFF(-1)-r2	CAAGATCTACCCATAGCCTCCTTTTTTTTTAAGATAAAG
cj1145c-ON-f1	CTTTATCTTAAAAAAAAAGGCGGAGTATGGGTAGATCTTG
cj1145c-ON-r1	CAAGATCTACCCATACTCCGCCTTTTTTTTTAAGATAAAG
cj1145c-r1E	GGGGGAATTCTCTCCTTTAAATCCATGGGC

cj1145c-f1E	GGGGGAATTCAGGTGCATAGGTCCACTGTC
cj1339c(flaA)-f1E	GGGGGAATTCTGCTACGCATCCTAATATCG
cj1339c(flaA)-r2E	GGGGAATTCCATAGGCTACTTGACCTATAG
cj1339c(flaA)-cat-r1	GTTTCCGGCGTTGTGGGAATCAAGCTCATCCATGAACTTG
cj1339c(flaA)-kan-r1	AATGGTTCGCTGGGTTTATCCAAGCTCATCCATGAACTTG
cj1420c-OFF(-1)-f2	CGTATATTGACAGGAGGCGGTATTTTACTGCGATTTGGA
cj1420c-OFF(-1)-r2	TCCAAATCGCAGTAAAATACCGCCTCCTGTCAATATACG
cj1420c-ON-f1	CGTATATTGACAGGTGGAGGCTATTTTACTGCGATTTGGA
cj1420c-ON-r1	TCCAAATCGCAGTAAAATAGCCTCCACCTGTCAATATACG
cj1420c-f1E	GGGGAATTCCATTTATCTTACATGATAGG
cj1420c-r1E	GGGGAATTCATACGCCCAGATATTATCCG
cj1421/22c-OFF(-1)-f2	GAACATAGACATAACGGAGGCGGTATATAGCATTA
cj1421/22c-OFF(-1)-r2	TAATGCTATATACCGCCTCCGTTATGTCTATGTTC
cj1421/22c-ON-f1	GAACATAGACATAACGGTGGAGGCTATATAGCATTA
cj1421/22c-ON-r1	TAATGCTATATAGCCTCCACCGTTATGTCTATGTTC
cj1421c-r1E	GGGGGAATTCCTAAATATCACCATCCAACTCCTTGC
cj1422c-f1E	GGGGAATTCATGTACCAAGTGGTAGTGGCTTGGG
cj1422c-f2E	GGGGAATTCGTTGGTGTTGTGCTTATTGGTG
cj1422c-r2E	GGGGAATTCCCAATACATCGTCTACTTTCACTTC
cj1426c-astA-r2	GCCCGCCAAAAGTGCCATACAAAGAGTTTTGCTAAGTCTATCAGTAATTAAGCCT GCATGTCCAC
cj1426c-OFF(-1)-f2	GTCGATAAATATGGAGGCGGGATGGATATCGTCC
cj1426c-OFF(-1)-r2	GGACGATATCCATCACCGCCTCCATATTTATCGAC

cj1426c-ON-f1	GTCGATAAATATGGTGGAGGCGATGGATATCGTCC
cj1426c-ON-r1	GGACGATATCCATCGCCTCCACCATATTTATCGAC
cj1426c-WT-f1	GTCGATAAATATGGGGGGGGGATGGATATCGTCC
cj1426c-WT-r1	GGACGATATCCATCCCCCCCCCATATTTATCGAC
cj1426c-f1E	GGGGAATTCTGGAAAATCAAGAGTCTTACCC
cj1426c-r1E	GGGGAATTCCATTCATAGCCCCGCCACTAGC
cj1429c-OFF(-1)-f2	GATGTATAATGGAGGCGGGATATGAGTGATATTAATGC
cj1429c-OFF(-1)-r2	GCATTAATATCACTCATATCCCGCCTCCATTATACATC
cj1429c-ON-f1	GATGTATAATGGTGGAGGCGATATGAGTGATATTAATGC
cj1429c-ON-r2	CGCCTCCACCATTATACATCACATAACCAC
cj1429c-f3E	GGGGAATTCGGTGGTATTCAACTCAAGGTGATCTTAGTG
cj1429c-r1E	GGGGAATTCATCCCATATTCTGAGATAGGACGCAAG
cj1437c-OFF(-1)-f3	CCTTAACTACTGGCGGAGGCGGATATTCAATGATTCTGT
cj1437c-OFF(-1)-r3	ACAGAATCATTGAATATACCGCCTCCGCCAGTAGTTAAGG
cj1437c-ON-f1	CCTTAACTACTGGCGGTGGAGGGATATTCAATGATTCTGT
cj1437c-ON-r1	ACAGAATCATTGAATATCCCTCCACCGCCAGTAGTTAAGG
cj1437c-r1E	GGGGAATTCGGTACAACAATAGAAACTTTTGGAG
cj1437c-f1E	GGGGAATTCGAAAGGATTAAATGGCAATTATC
cj1673c-f1000E	GGGGAATTCGCAACTAAGGTACTTTCATC
cj1673c-r1000E	GGGGAATTCGGTTTGATAAGTACAGCATTTG
cjj81176_1339-f1E	GGGGAATTCTCGGCAAGTACTCATCCTAG
cjj81176_1339-r1E	GGGGAATTCTTGGCCGTTATTATCACCATC
flaA81176-cat-r1	GTTTCCGGCGTTGTGGGAATCGAAATCCCATTTTAAATCC

flaA81176-kan-r1	AATGGTTCGCTGGGTTTATCCGAAATCCCATTTTAAATCC
kan-cj1339c(flaA)-f1	CCTAGATTTAGATGTCTAAAAAGCGCTGCAATATATACAAATCC
kan-flaA81176-f1	CCTAGATTTAGATGTCTAAAAAGCGCAATGGCTCAAGCAAATTC
pUCFa-176_1669-f1	GGAAGAGCACACGTCTGAACTCGCGAAGATGACGAAGGAGAAG
pUCFa-f1	TTTCCCTTAACGACGCTCTTCC
pUCFa-r1	GAGTTCAGACGTGTGCTCTTCC
rpsL(CJ0491)-StmR-F2	GGTGGTAGGGTAA<u>G</u>AGACTTACCAGGGG
rpsL(CJ0491)-StmR-R2	CCCCTGGTAAGTCT<u>C</u>TTACCCTACCACC
Used for MASC PCR	
astA-MASCR1	GCGGTCAAAGGAGACAATCCATAAGG
cj1139c-MASCmF2M	TATTAAAATTTTGGATATGGGTGG<u>C</u>Gc<u>A</u>
cj1139c-MASCmF3M	TATTAAAATTTTGGATATGGGTGG<u>A</u>Gc<u>C</u>
cj1139c-MASCR2	TATCTTTTTTGATTATTTTAGCCCACATTGTCC
cj1145c-MASCmF1M	TTTTAGATACAATTTACTTTATCTTAAAAAAAAAGG<u>C</u>Gc<u>A</u>
cj1145c-MASCmF2M	TTTTAGATACAATTTACTTTATCTTAAAAAAAAAGG<u>A</u>Gc<u>C</u>
cj1145c-MASCR1	GGATGTTGTGATTCTTGATTTTTTATTATCTTCATCCAC
cj1420c-MASCmF1M	GAATTTAATCGTATATTGACAGG<u>T</u>GG<u>A</u>Gc<u>C</u>
cj1420c-MASCmF2M	GAATTTAATCGTATATTGACAGG<u>A</u>Gc<u>C</u>
cj1420c-MASCR1	CTCCTTTTCAATTTCATCAAAAACCGAACC
cj1421c-MASCF1	TGAGGAATTGGTTTACATCAAGCAAC
cj1421c/22c-MASCmR1M	GAGTTTTTTAAATAATGCTATATA<u>G</u>CC<u>T</u>Cg<u>A</u>
cj1421c/22c-MASCmR2M	GAGTTTTTTAAATAATGCTATATACC<u>G</u>Cg<u>T</u>

cj1422c-MASCF4	ATGATTTTGATAGATACGGCACAGTAAATG
cj1426c-MASCmF1M	ATGCTTTATGTCGATAAATATGGTGGAGcC
cj1426c-MASCmF2M	ATGCTTTATGTCGATAAATATGGAGcC
cj1426c-MASCwF1M	CTTTATGTCGATAAATATGGGGGGGcG
cj1426c-MASCR1	TCCGTCTGACTGTCTTGTACACTTTC
cj1429c-MASCmF1M	GTGGTTATGTGATGTATAATGGTGGAGcC
cj1429c-MASCmF2M	GTGGTTATGTGATGTATAATGGAGcC
cj1429c-MASCR1	CCCATCTTGCTCCTCAGGATTGC
cj1437c-MASCF1	AGGCTTTTGCATTGGCGAGT
cj1437c-MASCmR1M	TTTGCTACAGAATCATTGAATATGCCTCgA
cj1437c-MASCmR2M	TTTGCTACAGAATCATTGAATATCCGCgT

[a]The single-underlined and double-underline nucleotides indicate EcoRI recognition sites and mutated sites, respectively. Lowercase letters in MASC primer sequences indicate mismatched bases that were used to improve the specificity by destabilizing the 3′-end of the non-allelic primer-template complex.
