# Supplementary material for: Stabilizing Genetically Unstable Simple Sequence Repeats in the Campylobacter jejuni Genome by Multiplex Genome Editing: a Reliable Approach for Delineating Multiple Phase-Variable Genes
Source: mBio. 2021 Aug 24;12(4):e01401-21. doi: 10.1128/mBio.01401-21 (PMC8437040; doi:10.1128/mBio.01401-21)
Supplement: TABLE S3 [file mbio.01401-21-st003.pdf]

Table S3. Specific combinations of donor DNA molecules and recipient strains used for natural transformation

Donor DNA (PCR fragment)	Recipient strain	Resulting strain
Δ*flaA*::*cat*-A	NCTC11168	SYC1001
Δ*flaA*::*kan*-A	NCTC11168	SYC1002
rpsL^{K88R}-8-A	NCTC11168	SYC1003
Δ*recA*::*cat*-A	NCTC11168	SYC1004
cj1426::*astA*, Δ*flaA*::*kan*-A	SYC1007	SYC1006
cj1426^{ON}::*astA*, Δ*flaA*::*cat*-A	NCTC11168	SYC1007
cj1426^{OFF}::*astA*, Δ*flaA*::*kan*-A	SYC1007	SYC1008
Δ*flaA*::*kan*-A (or Δ*flaA*::*cat*-A), *cj1139*^{OFF}, *cj1144*^{OFF}, *cj1420*^{OFF}, *cj1421*^{OFF}, *cj1422*^{OFF}, *cj1426*^{OFF}, *cj1429*^{OFF}, *cj1437*^{OFF}	NCTC11168	SYC1P000K
flaA^{+}-A	SYC1P000K	SYC1P000
Δ*flaA*::*cat*-A, *cj1420*^{OFF}, *cj1426*^{OFF}	SYC1P037	SYC1P001C
Δ*flaA*::*kan*-A, *cj1420*^{OFF}	SYC1P036C	SYC1P004K
Δ*flaA*::*cat*-A, *cj1420*^{OFF}	SYC1P037	SYC1P005C
Δ*flaA*::*kan*-A, *cj1426*^{OFF}	SYC1P036C	SYC1P032K
Δ*flaA*::*cat*-A, *cj1426*^{OFF}	SYC1P037	SYC1P033C
Δ*flaA*::*cat*-A, *cj1437*^{OFF}	SYC1P037	SYC1P036C
Δ*flaA*::*cat*-A, *cj1139*^{OFF}, *cj1144*^{OFF}, *cj1421*^{OFF}, *cj1422*^{OFF}, *cj1429*^{OFF}	SYC1P255	SYC1P037C
flaA^{+}-A	SYC1P037C	SYC1P037
Δ*flaA*::*kan*-A (or Δ*flaA*::*cat*-A), *cj1139*^{ON}, *cj1144*^{ON}, *cj1420*^{ON}, *cj1421*^{ON}, *cj1422*^{ON}, *cj1426*^{ON}, *cj1429*^{ON}, *cj1437*^{ON}	NCTC11168	SYC1P255K
flaA^{+}-A	SYC1P255K	SYC1P255
Δ*flaA*::*cat*-B	81-176	SYC2001
Δ*flaA*::*kan*-B	81-176	SYC2002
rpsL^{K88R}-8-B	81-176	SYC2003
Δ*recA*::*cat*-B	81-176	SYC2004
