# Supplementary material for: Stabilizing Genetically Unstable Simple Sequence Repeats in the Campylobacter jejuni Genome by Multiplex Genome Editing: a Reliable Approach for Delineating Multiple Phase-Variable Genes
Source: mBio. 2021 Aug 24;12(4):e01401-21. doi: 10.1128/mBio.01401-21 (PMC8437040; doi:10.1128/mBio.01401-21)
Supplement: TABLE S4 [file mbio.01401-21-st004.pdf]

Table S4. Primer mixes used for MASC PCR

Name	Size (bp)	ON/OFF detected	Phase
Mix ON1			
cj1422c-MASCF4 and cj1421c/22c-MASCmR1M	449	*cj1422*^{ON}	
cj1421c-MASCF1 and cj1421c/22c-MASCmR1M	319	*cj1421*^{ON}	
cj1437c-MASCF1 and cj1437c-MASCmR1M	250	*cj1437*^{ON}	
cj1145c-MASCmF1M and cj1145c-MASCR1	150	*cj1144*^{ON}	
Mix ON2			
cj1429c-MASCmF1M and cj1429c-MASCR1	400	*cj1429*^{ON}	
cj1426c-MASCmF1M and cj1426c-MASCR1	300	*cj1426*^{ON}	
cj1139c-MASCmF2M and cj1139c-MASCR2	200	*cj1139*^{ON}	
cj1420c-MASCmF1M and cj1420c-MASCR1	100	*cj1420*^{ON}	
Mix OFF1			
cj1422c-MASCF4 and cj1421c/22c-MASCmR2M	449	*cj1422*^{OFF}	
cj1421c-MASCF1 and cj1421c/22c-MASCmR2M	319	*cj1421*^{OFF}	
cj1437c-MASCF1 and cj1437c-MASCmR2M	250	*cj1437*^{OFF}	
cj1145c-MASCmF2M and cj1145c-MASCR1	150	*cj1144*^{OFF}	
Mix OFF2			
cj1429c-MASCmF2M and cj1426c-MASCR1	400	*cj1429*^{OFF}	

cj1426c-MASCmF2M and cj1426c-MASCR1	300	*cj1426*^{OFF}
cj1139c-MASCmF3M and cj1139c-MASCR2	200	*cj1139*^{OFF}
cj1420c-MASCmF2M and cj1420c-MASCR1	100	*cj1420*^{OFF}
