# Supplementary material for: Stabilizing Genetically Unstable Simple Sequence Repeats in the Campylobacter jejuni Genome by Multiplex Genome Editing: a Reliable Approach for Delineating Multiple Phase-Variable Genes
Source: mBio. 2021 Aug 24;12(4):e01401-21. doi: 10.1128/mBio.01401-21 (PMC8437040; doi:10.1128/mBio.01401-21)
Supplement: TABLE S5 [file mbio.01401-21-st005.pdf]

Table S5. Specific combinations of target genes and primers in PPT-Seq

Target gene	PCR primers	Sequencing primer
cj1139	cj1139c-f2E and cj1139c-r2E	cj1139c-MASCR2
cj1144	cj1145c-f1E and cj1145c-r1E	cj1145c-MASCR1
cj1420	cj1420c-f1E and cj1420c-r1E	cj1420c-MASCR1
cj1421	cj1422c-f2E and cj1422c-r1E	cj1421c-MASCF1
cj1422	cj1422c-f1E and cj1422c-r2E	cj1422c-MASCF4
cj1426	cj1426c-f1E and cj1426c-r1E	cj1426c-MASCR1
cj1429	cj1429c-f3E and cj1429c-r1E	cj1429c-MASCR1
cj1437	cj1437c-f1E and cj1437c-r1E	cj1437c-MASCF1
