# Supplementary material for: Stabilizing Genetically Unstable Simple Sequence Repeats in the Campylobacter jejuni Genome by Multiplex Genome Editing: a Reliable Approach for Delineating Multiple Phase-Variable Genes
Source: mBio. 2021 Aug 24;12(4):e01401-21. doi: 10.1128/mBio.01401-21 (PMC8437040; doi:10.1128/mBio.01401-21)
Supplement: TABLE S6 [file mbio.01401-21-st006.pdf]

Table S6. Primer sets used for allele-specific PCR and sequencing of *cj1426*::*astA* translational fusions

Reporter gene	Allele-specific PCR primers	Sequencing	
		PCR primers	Sequencing primer
cj1426::*astA*	cj1426c-MASCwF1M and astA-MASCR1	cj1426c-f1E and astA-MASCR1	astA-MASCR1
cj1426$_{\mathrm{ON}}$::*astA*	cj1426c-MASCmF1M and astA-MASCR1	cj1426c-f1E and astA-MASCR1	astA-MASCR1
cj1426$_{\mathrm{OFF}}$::*astA*	cj1426c-MASCmF2M and astA-MASCR1	cj1426c-f1E and astA-MASCR1	astA-MASCR1
